# Optimizing COVID-19 surveillance in long-term care facilities: a modelling study

**DOI:** 10.1186/s12916-020-01866-6

**Published:** 2020-12-08

**Authors:** David R. M. Smith, Audrey Duval, Koen B. Pouwels, Didier Guillemot, Jérôme Fernandes, Bich-Tram Huynh, Laura Temime, Lulla Opatowski, on behalf of the AP-HP/Universities/Inserm COVID-19 research collaboration

**Affiliations:** 1 Institut Pasteur, Epidemiology and Modelling of Antibiotic Evasion (EMAE), Paris, France; 2 Université Paris-Saclay, UVSQ, Inserm, CESP, Anti-infective evasion and pharmacoepidemiology team, Montigny-Le-Bretonneux, France; 3 Modélisation, épidémiologie et surveillance des risques sanitaires (MESuRS), Conservatoire national des arts et métiers, Paris, France; 4 Health Economics Research Centre, Nuffield Department of Population Health, University of Oxford, Oxford, UK; 5 The National Institute for Health Research (NIHR) Health Protection Research Unit in Healthcare Associated Infections and Antimicrobial Resistance, University of Oxford, Oxford, UK; 6 AP-HP, Paris Saclay, Public Health, Medical Information, Clinical Research, Le Kremlin-Bicêtre, France; 7 Clinique de soins de suite et réadaptation, Choisy-Le-Roi, France; 8 PACRI unit, Institut Pasteur, Conservatoire national des arts et métiers, Paris, France

**Keywords:** SARS-CoV-2, COVID-19, Testing, Infectious disease surveillance, Long-term care, Transmission dynamics, Computational modelling, Mathematical modelling, Contact network, Public health

## Abstract

**Background:**

Long-term care facilities (LTCFs) are vulnerable to outbreaks of coronavirus disease 2019 (COVID-19). Timely epidemiological surveillance is essential for outbreak response, but is complicated by a high proportion of silent (non-symptomatic) infections and limited testing resources.

**Methods:**

We used a stochastic, individual-based model to simulate transmission of severe acute respiratory syndrome coronavirus 2 (SARS-CoV-2) along detailed inter-individual contact networks describing patient-staff interactions in a real LTCF setting. We simulated distribution of nasopharyngeal swabs and reverse transcriptase polymerase chain reaction (RT-PCR) tests using clinical and demographic indications and evaluated the efficacy and resource-efficiency of a range of surveillance strategies, including group testing (sample pooling) and testing cascades, which couple (i) testing for multiple indications (symptoms, admission) with (ii) random daily testing.

**Results:**

In the baseline scenario, randomly introducing a silent SARS-CoV-2 infection into a 170-bed LTCF led to large outbreaks, with a cumulative 86 (95% uncertainty interval 6–224) infections after 3 weeks of unmitigated transmission. Efficacy of symptom-based screening was limited by lags to symptom onset and silent asymptomatic and pre-symptomatic transmission. Across scenarios, testing upon admission detected just 34–66% of patients infected upon LTCF entry, and also missed potential introductions from staff. Random daily testing was more effective when targeting patients than staff, but was overall an inefficient use of limited resources. At high testing capacity (> 10 tests/100 beds/day), cascades were most effective, with a 19–36% probability of detecting outbreaks prior to any nosocomial transmission, and 26–46% prior to first onset of COVID-19 symptoms. Conversely, at low capacity (< 2 tests/100 beds/day), group testing strategies detected outbreaks earliest. Pooling randomly selected patients in a daily group test was most likely to detect outbreaks prior to first symptom onset (16–27%), while pooling patients and staff expressing any COVID-like symptoms was the most efficient means to improve surveillance given resource limitations, compared to the reference requiring only 6–9 additional tests and 11–28 additional swabs to detect outbreaks 1–6 days earlier, prior to an additional 11–22 infections.

**Conclusions:**

COVID-19 surveillance is challenged by delayed or absent clinical symptoms and imperfect diagnostic sensitivity of standard RT-PCR tests. In our analysis, group testing was the most effective and efficient COVID-19 surveillance strategy for resource-limited LTCFs. Testing cascades were even more effective given ample testing resources. Increasing testing capacity and updating surveillance protocols accordingly could facilitate earlier detection of emerging outbreaks, informing a need for urgent intervention in settings with ongoing nosocomial transmission.

**Supplementary information:**

The online version contains supplementary material available at 10.1186/s12916-020-01866-6.

## Background

From nursing homes to rehabilitation hospitals, long-term care facilities (LTCFs) worldwide are hotspots for outbreaks of coronavirus disease 2019 (COVID-19) [1]. LTCF patients (or residents) require continuing care, live in close proximity to one another, and are typically elderly and multimorbid, placing them at elevated risk of both acquiring severe acute respiratory syndrome coronavirus 2 (SARS-CoV-2, the virus) and suffering severe outcomes from COVID-19 (the disease) [2–4]. Healthcare workers (HCWs) are also susceptible to infection and, amidst imperfect hygiene and infection prevention measures, potentially transmit the virus through necessary daily interactions with both patients and staff [1, 5]. Although the full extent of the ongoing pandemic is unclear and ever-evolving, LTCFs have and continue to bear a disproportionate burden of SARS-CoV-2 infection and COVID-19 mortality [3, 6, 7]. Across Europe, for instance, LTCFs have accounted for an estimated 30–60% of all COVID-19 deaths as of June 2020 [8].

Effective COVID-19 surveillance is essential for timely outbreak detection and implementation of necessary public health interventions to limit transmission, including case isolation, contact tracing and enhanced infection prevention [9–11]. The current gold-standard diagnostic test for active SARS-CoV-2 infection is reverse transcriptase polymerase chain reaction (RT-PCR), typically performed on clinical specimens from nasopharyngeal swabs [12]. Though sensitive and highly specific, RT-PCR is relatively resource intensive, must be outsourced for institutions lacking on-site infrastructure, and is widely subject to shortages and specific usage guidelines. For instance, a common practice in LTCFs in France, the Netherlands, the UK, the USA, and elsewhere has been to restrict testing to individuals presenting with characteristic COVID-19 symptoms [4, 13–15]. Yet symptomatic infections represent just the tip of the iceberg: many infections cause no or only mild symptoms, produce high quantities of virus in the absence of symptoms, and experience relatively long delays until symptom onset [16–19]. Silent transmission from asymptomatic and pre-symptomatic infections is a known driver of COVID-19 outbreaks [20, 21], with non-symptomatic cases acting as Trojan Horses, unknowingly introducing the virus into healthcare institutions and triggering nosocomial spread [8, 22, 23].

Insufficient surveillance systems, including those lacking testing capacity or relying only on symptoms as indications for testing, have been identified as aggravating factors for COVID-19 outbreaks in LTCFs [8, 16, 24–27]. Various surveillance strategies have been proposed to optimize testing while accounting for the particular transmission dynamics of SARS-CoV-2, including randomly testing HCWs, testing all patients upon admission, and universal or serial testing [28–30]. Yet COVID-19 surveillance is limited in practice by available testing capacity and health-economic resources, particularly for institutions in low- and middle-income settings [31, 32]. In light of testing shortages, group testing (sample pooling, combining clinical specimens from multiple individuals into a single biological sample for a single RT-PCR test) has garnered attention as a diagnostically sensitive and resource-efficient alternative to individual-based testing [33–38].

In order to mitigate and prevent future nosocomial outbreaks, there is an urgent need to optimize COVID-19 surveillance in long-term care settings, taking into account both the unique epidemiological characteristics of SARS-CoV-2 and limited availability of testing resources [1]. Here, we investigated the efficacy, timeliness, and resource efficiency of a range of COVID-19 surveillance strategies using simulations from a dynamic SARS-CoV-2 transmission model that uses detailed inter-individual contact data to describe interactions between patients and staff in long-term care.

## Methods

### Simulating COVID-19 outbreaks in long-term care

We simulated nosocomial outbreaks of COVID-19 using a dynamic, stochastic, individual-based Susceptible Exposed Infectious Recovered (SEIR) model of SARS-CoV-2 transmission coded in C++ [39]. The model is described fully in Additional File 1 using the Overview, Design concepts, and Details (ODD) protocol for individual-based modelling [12, 18, 38–57]. The goals of our model are to simulate (i) dynamic inter-individual contacts among patients and staff in an LTCF setting, (ii) transmission of SARS-CoV-2 along simulated contact networks, and (iii) clinical progression of COVID-19 among individuals infected with SARS-CoV-2. Throughout, COVID-19 refers to any case of SARS-CoV-2 infection, and not only symptomatic cases.

### Characterizing LTCF structure, demographics, and inter-individual contact behaviour

**Fig. 1:**
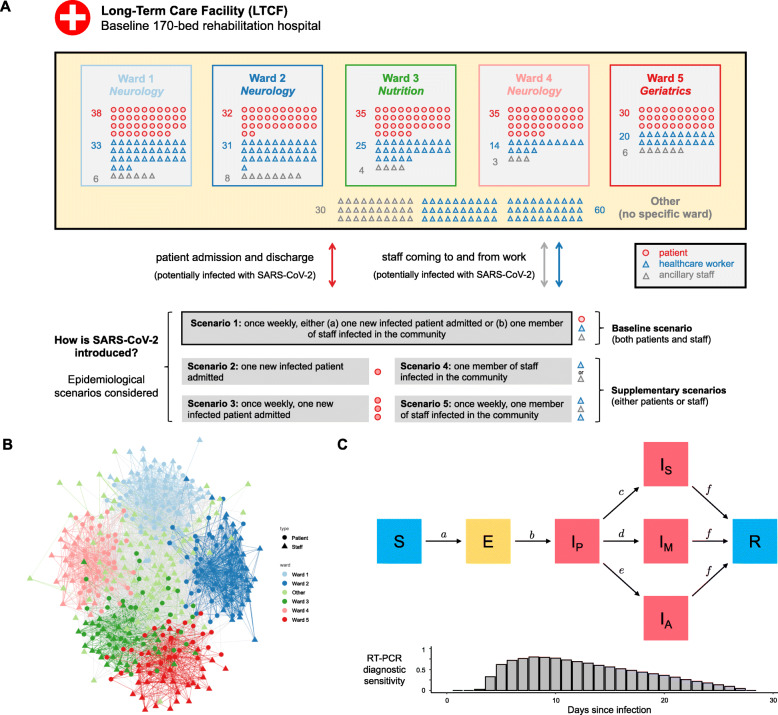
Characteristics of the SARS-CoV-2 transmission model. **a** A diagram of the baseline LTCF, showing the average weekly number of patients and staff in each ward, including “Other” staff not primarily in any one specific ward. Below the LTCF is a description of the epidemiological scenarios considered for how SARS-CoV-2 was introduced into the LTCF. **b** A snapshot of the simulated dynamic contact network, showing all patients (PA, circles) and staff (PE, triangles) present in the baseline LTCF as nodes, and inter-individual contacts aggregated over one randomly selected day as edges. Nodes and edges are coloured by ward, with grey edges representing contacts across wards. **c** A diagram of the modified SEIR process used to characterize COVID-19 infection (S, susceptible; E, exposed; I_P_, infectious pre-symptomatic; I_A_, infectious asymptomatic; I_M_, infectious with mild symptoms; I_S_, infectious with severe symptoms; R, recovered), with transitions between states *a* to *f* (see Additional File 1: Table S1). Below, diagnostic sensitivity of RT-PCR for detecting SARS-CoV-2 in a true positive specimen was modelled as a function of time since infection

We used data from the i-Bird study to inform the population structure and dynamic inter-individual contact network used in our model. The i-Bird study has been described elsewhere [41, 58]; briefly, close-proximity interactions were measured every 30 s by sensors worn by all patients and staff over a 17-week period in 2009 in a rehabilitation hospital in northern France. There were 170 patient beds across the five wards of this LTCF, and staff were distributed across 13 categories of employment, grouped here as HCWs (caregiver, nurse, physiotherapist, occupational therapist, nurse trainee, physician, and hospital porter) or ancillary staff (hospital services, administration, other rehabilitation staff, management, logistical staff, and activity coordinator/hairdresser) (Fig. 1a).

This population structure was used in our model, but a novel contact network was simulated to account for missing data resulting from imperfect sensor compliance in the raw contact network (Fig. 1b; described in Additional File 1). Real patient admission and staff timesheet data were used to determine who was present in the LTCF: there were on average 170 patients and 240 staff present each week, stratified by ward and type of individual (e.g. patient, nurse, logistical staff) in Additional File 2: Table S1. The probability of coming into contact with another individual at each model time-step (30 s) was estimated from the raw contact data and stratified by hour of the day (e.g. 8:00:00–8:59:30, 9:00:00–9:59:30), day of the week (weekday vs. weekend), ward and type of individual. At each time-step, new contacts were simulated based on these probabilities, and contact durations were drawn stochastically from log-normal distributions stratified by the same variables. Contact behaviours were comparable between the raw and simulated networks, and fidelity of the simulated network has been validated previously by its ability to reproduce transmission dynamics from a real outbreak of methicillin-resistant *Staphylococcus aureus* in this LTCF [55, 58].

### Characterizing SARS-CoV-2 transmission and COVID-19 infection

Parameter estimates from the literature were used to characterize SARS-CoV-2 transmission and clinical progression of COVID-19 (see Additional File 1: Table S2). We assumed that susceptible patients and staff could become infected with SARS-CoV-2 if in direct contact with an infectious individual, with the probability of transmission depending on the duration of contact. Owing to a lack of data from healthcare settings, SARS-CoV-2 transmissibility was estimated using data from the community (see Additional File 1). Assuming *R*_0_ = 3 in France prior to lockdown [44], and using pre-pandemic inter-individual contact data [45], we derived a transmission probability per minute spent in contact with an infectious individual *p* = 0.14%. Under baseline assumptions in our simulated LTCF, this estimate for *p* resulted in a mean *R*_0_ = 4.04 (Additional File 2: Fig. S1). This is consistent with the finding that, for any given value of *p*, *R*_0_ is expected to vary between community and healthcare settings because of fundamental differences in inter-individual contact behaviour [43]. However, to reflect considerable uncertainty in the true value of *p*, we considered extreme values in sensitivity analysis (*p* = 0.07%, 0.28%).

Clinical progression of COVID-19 was characterized by a modified SEIR process, with (i) a non-infectious exposed period of 2–5 days, (ii) an infectious pre-symptomatic period of 1–3 days, (iii) an on-average 7-day infectious “symptomatic” period with three levels of symptom severity (severe, mild or asymptomatic), and (iv) eventual recovery with full immunity (Fig. 1c). Together, (i) and (ii) amount to an incubation period of 3–8 days including a 1–3-day window of pre-symptomatic transmission, consistent with estimates used elsewhere [18, 19, 59]. For (iii), we assumed that 70% of infected individuals develop clinical symptoms [51, 60, 61], 20% of which develop severe/critical symptoms [52]. Durations for each stage of infection were drawn probabilistically from their respective distributions for each infection. We assumed no difference in average time to symptom onset for mild symptomatic and severe symptomatic cases. As surveillance strategies were evaluated only for detection of outbreaks, death and potential long-term clinical outcomes were not explicitly simulated. Each simulation began by introducing one non-symptomatic index case (either exposed, pre-symptomatic or asymptomatic) into the facility on the first day of each simulation (*t* = 0). In the baseline scenario, both patients and staff introduced SARS-CoV-2 into the LTCF.

### Measuring outbreaks

Simulated COVID-19 outbreaks were described using various outcome measures, including infection incidence, infection prevalence, case distribution (proportion of infections among patients, HCWs, and ancillary staff), outbreak size (cumulative number of cases after 12 weeks of unmitigated transmission, the full duration of each outbreak simulation), and outbreak size upon first presentation of COVID-19 symptoms.

### Developing a COVID-19 surveillance algorithm

We developed a stochastic surveillance algorithm to evaluate a range of surveillance strategies for their efficacy, timeliness, and resource-efficiency in detecting COVID-19 outbreaks. Strategies varied according to who received conventional nasopharyngeal swabs and RT-PCR tests, and with what priority. Demographic and clinical indications were used to administer swabs and tests, beginning on the first day of each outbreak simulation and assuming a daily maximum testing capacity ranging from 1 to 32 tests/day. We assumed a 24-h lag from swab to test result, perfect specificity (true negative rate) and imperfect sensitivity (true positive rate). Using data from a meta-analysis of RT-PCR for detection of SARS-CoV-2 in upper respiratory samples, sensitivity was modelled as a function of time since infection, ranging from 0% on the first 2 days after infection, to 33% after 4 days, peaking at 80% after 8 days, and gradually decreasing thereafter (Additional File 1: Fig. S1) [54]. Each test result was determined stochastically according to these parameters. The algorithm is described in further detail in Additional File 1.

### Surveillance strategies considered

Four types of surveillance were evaluated: (i) testing individuals with particular indications, (ii) random testing, (iii) testing cascades, and (iv) group testing (Table 1). Each was further divided into distinct surveillance strategies. For (i), three indications were considered: presentation of severe COVID-like symptoms (reference strategy), presentation of any COVID-like symptoms, or new admission to the LTCF. For (ii), tests were randomly distributed among patients, HCWs, or all patients and staff. In contrast to (i) and (ii), strategies (iii) and (iv) were conceived as hierarchical testing protocols, in which individuals presenting with severe COVID-like symptoms were always tested first to reflect their clinical priority. Remaining tests were subsequently allocated via cascades (iii) or as a single group test (iv).

**Table 1:** Surveillance strategies evaluated for detection of COVID-19 outbreaks in a LTCF. Strategies differ in how swabs and tests are apportioned to patients and staff. Arrows (→) indicate order of priority for testing cascades. *Test*, RT-PCR test; *swab*, nasopharyngeal swab; *symptoms*, COVID-like symptoms; *admission*, arrival of new patient to the LTCF

Surveillance type	Description	Surveillance strategy	Daily testing capacity always reached?
**Single indication**	Administer tests to any individuals indicated for testing, up to the daily testing capacity. If the number of individuals indicated exceeds the number of tests available, select randomly among them.	Symptoms (severe) *[reference strategy]*	No
		Symptoms (any)	No
		Admission	No
**Random**	Each day, randomly administer tests to individuals in a particular demographic group.	Random (patients)	Yes
		Random (HCWs)	Yes
		Random (all: patients, HCWs and ancillary staff)	Yes
**Cascade**	A combination of indications and random testing. First, use indications to administer tests according to a given order of priority. Then, if any tests remain, distribute them randomly among patients not otherwise indicated for testing.	Symptoms (severe) → Symptoms (mild) → Random (patients)	Yes
		Symptoms (severe) → Symptoms (mild) → Admission → Random (patients)	Yes
		Symptoms (severe) → Admission → Random (patients)	Yes
		Symptoms (severe) → Admission → Symptoms (mild) → Random (patients)	Yes
**Group testing**	Classic two-stage sample pooling, modified to account for clinical urgency of severe COVID-19. First, administer individual tests to any patients or staff presenting with severe symptoms. Then, if at least one test remains, pool clinical specimens together and run one test across this group sample. If the test result is positive, individually re-swab and re-test all included individuals to identify cases. The maximum number of samples per group test was varied from 2 to 64.	Symptoms (any)	No
		Admission	No
		Random (patients) (always maximizes number of specimens per group test)	No
		Random (HCWs) (always maximizes number of specimens per group test)	No

For (iii), testing cascades were conceived as mixed testing strategies combining (i) and (ii), in which multiple indications were considered simultaneously but ordered according to their perceived clinical priority. If there were more tests available than individuals indicated for testing, remaining tests were distributed randomly among remaining patients, such that cascades always maximized daily testing capacity. For (iv), clinical specimens from individual swabs were pooled together and tested as one, up to a maximum of 32 swabs per test in the baseline analysis. Various studies have demonstrated the efficacy of this method for SARS-CoV-2 detection, with sufficient diagnostic accuracy to detect a single SARS-CoV-2-positive specimen pooled with 30+ negative specimens [38, 62, 63]. Yet diluting positive specimens nonetheless reduces the concentration of viral RNA in the sample, which should reduce sensitivity of a group test compared to an individual test of the same positive specimen [64]. We estimated a 0.7% reduction in test sensitivity per additional negative specimen added to a group sample when assuming a typical RT-PCR Cycle threshold cut-off (Ct = 40), and for sensitivity analysis estimated a faster rate (1.3%) corresponding to a stricter threshold (Ct = 35) (see Additional File 1). Various group testing procedures have been proposed elsewhere [33, 34, 37, 65, 66]; here we evaluated a simple two-stage “Dorfman” protocol that does not require additional investment or infrastructure, but which requires all individuals included in the initial group test to be re-swabbed and re-tested individually upon a positive group test result in order to determine which individual(s) is (are) infected [35, 67].

### Administering swabs and tests

Each swab resulted in one RT-PCR test, except for group testing strategies, in which multiple swabs were combined per test. Admission-based tests were administered upon a patient’s arrival to the LTCF, and symptom-based tests were administered on the first day that symptoms appeared. We further assumed that no individuals refused swabbing/testing. Clinically, COVID-19 can resemble other common acute respiratory infections, such that individuals not infected with SARS-CoV-2 can nonetheless present with COVID-like symptoms and be indicated for symptom-based testing [68]. We used influenza-like illness as a proxy for COVID-like symptoms of aetiologies other than SARS-CoV-2. The daily incidence rate of influenza-like illness in our LTCF setting (1.1%) was calculated using data from 2008 to 2017 from French emergency departments (OSCOUR network) as the daily incidence rate of influenza-like illness among older adults (50–99 years) [53]. As with actual COVID-19, we assumed that 20% of these individuals also present with severe symptoms.

### Surveillance outcomes evaluated

Surveillance strategies were evaluated for their ability to detect COVID-19 outbreaks using four primary outcome measures: first, the probability of detecting an outbreak (i) at any time *t* from the index case at *t* = 0, (ii) prior to any secondary cases (interpreted as the probability of detecting the index case before any nosocomial transmission), or (iii) prior to first presentation of COVID-19 symptoms. Second, detection lag, the number of days from the index case to outbreak detection (first positive test result). For group testing, this was taken as the date of the first positive group test result (first round of testing) and not the date of subsequent case identification (second round). We defined a maximum detection lag of 22 days, after which all outbreaks were assumed to be detected regardless of the surveillance strategy used. Third, outbreak size upon detection, the cumulative number of cases at first positive test result. Fourth, the total number of (i) nasopharyngeal swabs used and (ii) RT-PCR tests conducted until outbreak detection. Here, for group testing, this does include the second round of testing, i.e. resources required to individually re-swab and re-test all individuals included in the initial positive group test.

### Measuring surveillance efficiency

From a health-economic perspective, an efficient use of healthcare resources is one that yields better health outcomes than alternative uses of the same resources [69]. Efficiency can be measured using incremental analysis, in which the additional cost of a particular intervention compared to a reference baseline is scaled by its additional health benefit [70]. This is traditionally expressed as the incremental cost-effectiveness ratio using monetary costs and standardized units of health benefit (e.g. quality-adjusted life-years gained). To report on efficiency in terms of the surveillance cost and benefit outcomes measured in this study, we defined a similar metric, the incremental efficiency ratio (IER),

$$ \mathrm{IER}=\frac{{\left(\mathrm{surveillance}\ \mathrm{resource}\ \mathrm{use}\right)}_S-{\left(\mathrm{surveillance}\ \mathrm{resource}\ \mathrm{use}\right)}_R}{{\left(\mathrm{surveillance}\ \mathrm{outcome}\right)}_S-{\left(\mathrm{surveillance}\ \mathrm{outcome}\right)}_R}, $$

for each surveillance strategy *S* relative to the reference *R*. Efficiency results were calculated using the IER as the number of additional swabs and tests required per 1-case reduction in outbreak size upon detection (for simplicity, reported as mean additional swabs and tests used per case averted). Here, we took the perspective of an LTCF with a reference strategy of only testing individuals with severe COVID-like symptoms.

### Uncertainty and sensitivity analysis

We ran a range of sensitivity analyses to account for uncertainty in (i) how SARS-CoV-2 was introduced into the LTCF, (ii) LTCF size and structure, (iii) transmissibility of SARS-CoV-2, and (iv) diagnostic sensitivity of RT-PCR for individual samples and (v) for group samples (see assumptions in Additional File 1). For each scenario, 100 epidemics were simulated using the transmission model. For each simulated epidemic, the surveillance algorithm was run 100 times across six testing capacities (1, 2, 4, 8, 16 or 32 tests/day), for a total 60,000 stochastic simulations for each surveillance strategy and each scenario. Across all scenarios, we also varied the maximum number of swabs potentially included per group test (2, 4, 8, 16, 32, or 64 swabs/test). Outcomes were only evaluated for epidemic simulations that resulted in nosocomial outbreaks, defined as simulations with ≥ 1 new case of COVID-19 within 21 days of the initial index case. Unless stated otherwise, outcome measures are reported as median values across all simulations, with uncertainties expressed as 95% uncertainty intervals, i.e. outcomes from the 2.5th and 97.5th percentiles. Supplementary results can be found in Additional File 2.

## Results

### SARS-CoV-2 spreads quickly, but COVID-19 symptoms lag behind

**Fig. 2:**
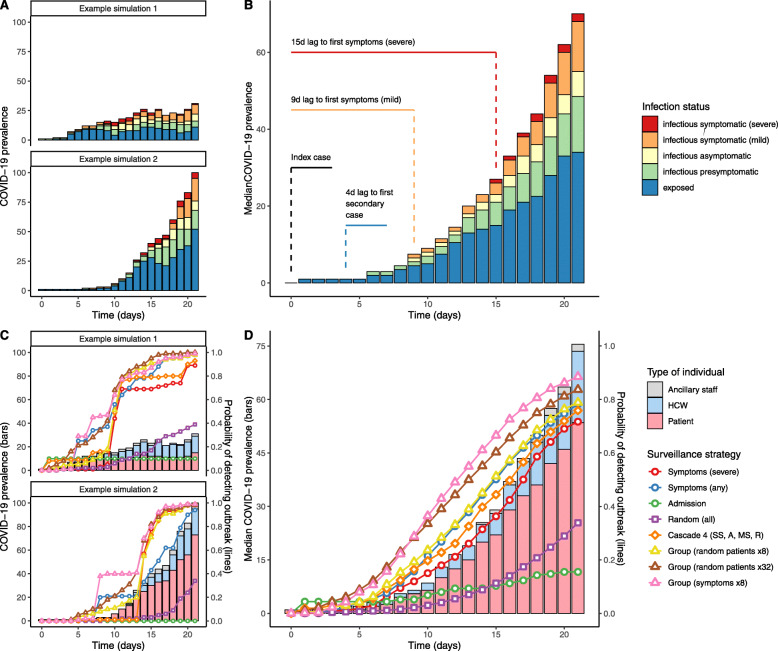
Epidemic curves of COVID-19 infection resulting from random introductions of SARS-CoV-2 into a 170-bed LTCF. Symptomatic cases represent just the “tip of the iceberg” in nascent outbreaks. **a** Two examples of epidemic simulations, demonstrating variation in outbreak velocity and lags until first onset of COVID-19 symptoms. **b** The median epidemic curve across all simulations for the baseline scenario, with dotted lines demarcating median time lags to selected events. Bars represent the median number of individuals in each infection class over time, and do not necessarily total to the median number infected (e.g. there is a median 1 infection at *t* = 0 but a median 0 infections in each class, as each index case had an equal 1/3 probability of being exposed, pre-symptomatic or asymptomatic). For the same simulation examples (**c**) and median (**d**), the probability of detecting outbreaks varied over time for different surveillance strategies (coloured lines), depending on how many, and which types of individuals became infected over time (vertical bars); here, testing capacity = 1 test/day

SARS-CoV-2 spread quickly, but with a great degree of stochasticity upon its random introduction to simulated LTCFs (Fig. 2, Additional File 2: Fig. S2). After 3 weeks of unmitigated transmission, a cumulative 86 (95% uncertainty interval 6–224) individuals were infected, predominantly other patients (median 72%), then HCWs (25%), and ancillary staff (3%) (Additional File 2: Table S2). Outbreaks were characterized by a median lag of 9 (2–24) days between the non-symptomatic index case entering the LTCF and first presentation of mild COVID-19 symptoms among any patient or staff in the facility (Additional File 2: Table S3). By the time symptoms emerged, an additional 5 (0–29) individuals had acquired SARS-CoV-2 but were not (yet) showing symptoms (Additional File 2: Table S4). Lags were longer for first presentation of severe COVID-19 symptoms (15 days from index case, 4–28), coinciding with a greater cumulative number of secondary infections (25, 0–101).

### Less effective surveillance strategies allow infections to accumulate

Surveillance strategies varied in their ability to detect emerging COVID-19 outbreaks. Surveillance efficacy depended on the stochastic nature of outbreaks, including how many, and which types of individuals became infected over time (Fig. 2c, d). Outbreaks grew exponentially at their outset, so delaying outbreak detection by just 1 or 2 days potentially coincided with tens more infections (Additional File 2: Fig. S3). Five days from the index case entering the LTCF, only 2 (1–12) individuals were infected; after 10 days, 9 (1–44) were infected; and after 15 days, 36 (2–124) patients and staff were infected.

### Optimal surveillance depends on daily testing capacity

**Fig. 3:**
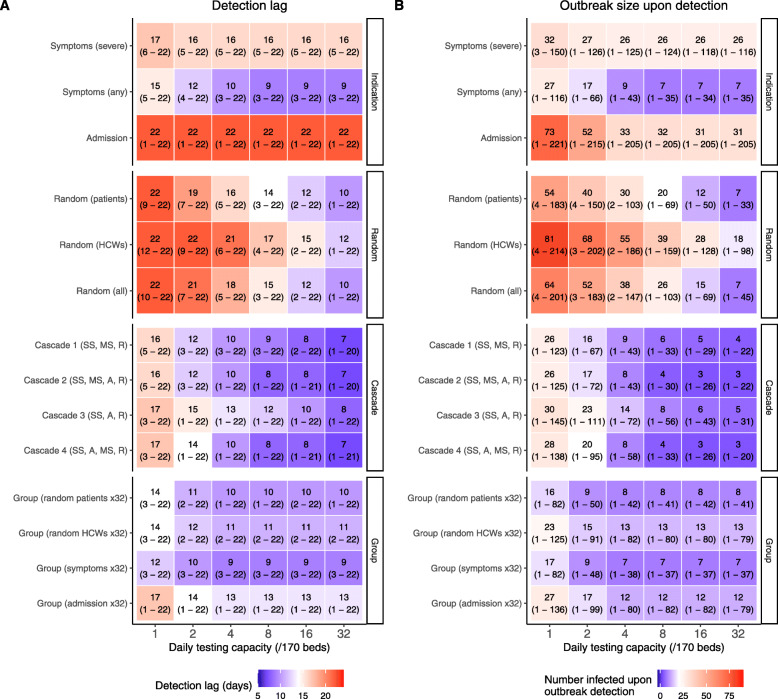
Test more to detect outbreaks sooner. **a** Median lags to outbreak detection (95% uncertainty interval) and **b** corresponding median outbreak sizes upon detection (95% uncertainty interval) are shown for each surveillance strategy (*y*-axis) as a function of the daily testing capacity (*x*-axis). Group testing strategies assume a maximum of 32 swabs per test. For both cascades and group testing, individual tests were always reserved for individuals with severe COVID-like symptoms; remaining tests were then distributed according to cascades or as a single group test. SS, severe symptoms; MS, mild symptoms; A, admission; R, random patients

Across all testing capacities, only testing individuals with severe COVID-like symptoms was among the least effective surveillance strategies considered (Fig. 3). This “reference” strategy took a median 16–17 days to detect outbreaks and had a 2–4% probability of detecting the initial index case prior to any secondary cases (Additional File 2: Fig. S4). Instead of only severe symptoms, testing individuals with any COVID-like symptoms was more effective, taking 9–15 days to detect outbreaks, with a 3–14% probability of detecting index cases prior to any secondary cases. Only testing patients at admission was overall ineffective by right of detecting neither staff index cases nor ongoing outbreaks already underway in the LTCF, resulting in long median delays to outbreak detection despite comparatively high probabilities of detecting COVID-19 prior to any secondary cases (10–33%). In the scenario where only new patients introduced SARS-CoV-2 into the LTCF, there was a 34% probability of detecting the index case when testing all patients upon admission, or 66% in a sensitivity analysis considering higher and more stable RT-PCR sensitivity. For random testing strategies, surveillance was highly ineffective when few tests were available, but increasingly effective at higher testing capacities. Conversely, for indication-based strategies, efficacy plateaued when capacity exceeded the number of individuals indicated for testing (Additional File 2: Fig. S4).

At high testing capacity (16–32 tests/day, ≈ 9–19 tests/100 beds/day), testing cascades were the most effective surveillance strategies. The four cascades considered here detected outbreaks within a median 7–10 days, coinciding with just 3–6 COVID-19 infections among all patients and staff. Cascades had a 19–36% chance of detecting outbreaks prior to any secondary cases, a 26–46% chance prior to the emergence of any COVID-19 symptoms, and a 64–85% chance prior to severe COVID-19 symptoms. Cascades that included both new patient admission and presentation of any COVID-like symptoms as indications for testing were most effective.

At low testing capacity (1 or 2 tests/day, ≈ 0.6–1.2 tests/100 beds/day), group testing was the most effective form of surveillance considered. Compared to the reference (16–17 days) and cascades (16–17 days), outbreaks were detected within 11–14 days (coinciding with a cumulative 9–16 infections) when pooling random patients, or 12–14 days (15–23 infections) when pooling random HCWs. At this low capacity, it was also more effective to pool symptomatic individuals in group tests (10–12 days, 9–17 infections) than to test them individually (12–15 days, 17–27 infections) because individuals with non-COVID but COVID-like symptoms were also “in competition” for limited tests. Compared to the baseline protocol, which assumed a maximum of 32 swabs/test, group testing was less effective given fewer swabs per test, despite potentially higher test sensitivity. For example, when pooling randomly selected patients in daily group tests, outbreaks were detected within 11–14 days at 32 swabs/test, 12–15 days at 8–16 swabs/test, and 14–17 days at 2–4 swabs/test. In a sensitivity analysis considering a stricter RT-PCR diagnostic threshold (Ct = 35 instead of 40), group testing was most effective at 16–32 swabs per test (12–15 days), but declined substantially for greater group sample sizes (16–17 days at 64 swabs/test) (Additional File 2: Fig. S5).

### Group testing symptomatic individuals is the most efficient use of both swabs and tests

**Fig. 4:**
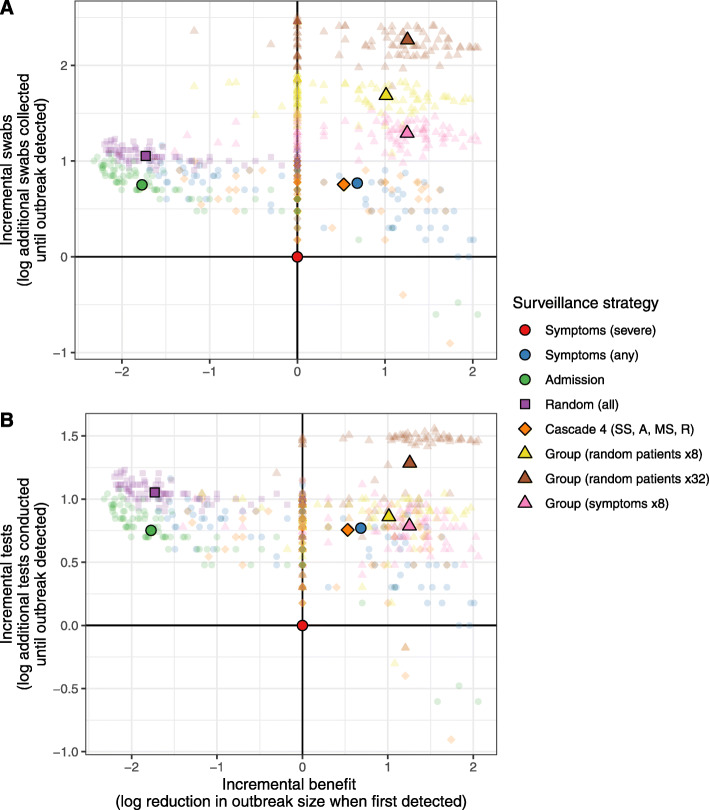
Incremental efficiency plots for selected surveillance strategies relative to a reference strategy of only testing individuals with severe COVID-like symptoms. Here, improvement in COVID-19 surveillance (*x*-axis) is balanced against additional nasopharyngeal swabs used (*y*-axis for **a**) and additional RT-PCR tests conducted (*y*-axis for **b**) until outbreaks were detected. Both axes are log_10_-adjusted. For both panels, daily testing capacity is fixed at 1 test/day (for higher testing capacities, see Additional File 2: Fig. S7). Small translucent points represent median outcomes across 100 surveillance simulations for each simulated outbreak, and larger opaque points represent mean of medians across all outbreaks

Surveillance strategies varied considerably in their use of testing resources (Additional File 2: Fig. S6) and in their efficiency for improving COVID-19 outbreak detection relative to the reference strategy (Fig. 4). The reference used the fewest swabs and tests, on average < 1/day regardless of the assumed daily testing capacity (owing to a low daily incidence of severe COVID-like symptoms). At high testing capacity (16–32 tests/day), the high incremental efficacy of cascades (outbreak detection a mean 5–8 days earlier than the reference, prior to 22–27 additional infections) resulted from extensive resource use (104–276 additional tests and swabs), for mean efficiencies of 4.0–11.2 additional swabs and tests per case averted. Although simply testing all patients and staff with any COVID-like symptoms was less effective than using testing cascades, it was a more efficient means to improve surveillance (mean 1.3 additional tests per case averted).

Group testing strategies were generally efficient with respect to tests, but used highly variable numbers of swabs to detect outbreaks. At high swabbing capacity (16–32 swabs/group test, ≈9–19 swabs/100 beds/day) and across all testing capacities, pooling randomly selected patients used a mean 11–38 excess tests to detect outbreaks 2–4 days earlier and prior to 14–21 additional infections (0.8–1.8 additional tests per case averted), but a median 94–384 additional swabs (6.7–18.7 additional swabs per case averted). Pooling the same number of randomly selected HCWs was less efficient than pooling patients (detection = 1–4 days earlier, prior to 6–14 infections; efficiency = 1.3–2.8 additional tests and 15.9–27.5 additional swabs per case averted). By contrast, for all scenarios and testing capacities considered, pooling individuals with any COVID-like symptoms was among the most efficient strategies in terms of both swabs and tests. In the most resource-limited scenarios (1–2 tests, ≈ 0.6–1.2 tests/100 beds/day; 2–8 swabs per group test, ≈ 1–5 swabs/100 beds/day), this was both the most effective means to detect COVID-19 outbreaks and the most efficient means to improve surveillance from the reference (detection = 1–6 days earlier, prior to 11–22 additional infections; efficiency = 0.3–0.6 additional tests and 1.0–1.3 additional swabs per case averted).

## Discussion

The ongoing COVID-19 pandemic continues to devastate LTCFs worldwide, with high rates of mortality among particularly frail and elderly patients, and high rates of infection among patients and staff alike [3, 5, 6, 8]. This motivates a need for timely and efficient surveillance strategies that optimize limited testing resources to detect emerging outbreaks as quickly as possible. We used an individual-based transmission model to simulate COVID-19 outbreaks in LTCF settings and evaluated a range of testing strategies for their efficacy and efficiency in detecting these outbreaks across various epidemiological assumptions and scenarios.

Our findings suggest that LTCFs can detect emerging COVID-19 outbreaks most quickly by using testing cascades, provided that they have substantial daily testing capacity (on the order of at least 1 test/10 beds/day). The most effective cascades considered multiple indications, including both COVID-like symptoms and patient admission, and detected outbreaks days ahead of traditional symptom-based screening and prior to the accumulation of additional infections. By extension, cascades had the greatest probability of identifying non-symptomatic cases, a known challenge for COVID-19 surveillance in real LTCF settings [1]. These findings held in sensitivity analyses considering outbreaks in a smaller, 30-bed geriatric LTCF (Additional File 2: Fig. S8), as well as when halving or doubling SARS-CoV-2 transmissibility (Additional File 2: Figs. S9, S10). Although only a select few indications were considered in the present study, LTCFs may consider a wider range of known risk factors for SARS-CoV-2 acquisition in their own cascades to maximize the probability of detecting emerging outbreaks before widespread transmission.

COVID-19 surveillance was less effective in resource-limited settings because of an inability to regularly test large numbers of patients and staff. In our analysis, group testing was the most effective means of COVID-19 surveillance under limited testing capacity, and across all epidemiological scenarios and capacities was the most resource-efficient means to improve surveillance with respect to a “bare minimum” reference of only testing individuals with severe COVID-like symptoms. Even when assuming strict diagnostic cut-offs in sensitivity analysis, group testing strategies remained effective up to a maximum of 32 swabs per test (Additional File 2: Fig. S5). This broadly agrees with modelling results suggesting that group testing could be cost-effective for screening in large populations, as well as empirical evidence for the efficiency of group testing for COVID-19 surveillance in nursing homes [33, 71]. As with cascades, LTCFs that conduct group testing may consider a wider range of indications than was possible to include in this study, in order to maximize the probability of including potentially infected patients and staff in routine group tests. These findings reinforce current guidance from the World Health Organization, endorsing sample pooling to increase COVID-19 diagnostic capacity when testing demand outstrips supply, but cautioning against its use for contact tracing or in high-prevalence settings [63]. This is consistent with its implementation in the present study, as a means of surveillance in resource-limited long-term care settings without known active cases, but which are nonetheless susceptible to outbreaks.

Our analysis was limited to classical two-stage group testing, initially proposed by Dorfman in 1943 for syphilis screening among World War II soldiers [67], in which all individuals in a positive group test are individually re-tested to determine who is infected. This is regarded as the most straightforward approach [35], and we conservatively assumed re-swabbing in addition to re-testing of all individuals in a positive group test to account for potential logistical challenges of storing and maintaining large numbers of swabs for re-testing. Various alternative group testing strategies have been proposed and implemented elsewhere, including the use of simultaneous multi-pool samples, non-adaptive pooling schemes, and others [35, 37, 65, 66]. These have the advantage of not requiring separate re-testing of all individuals in a positive group test and are hence more efficient in terms of the number of tests required for case identification. However, these strategies may also require additional testing infrastructure and expertise, which may be cost-prohibitive for the resource-limited settings that may benefit most from group testing in the first place. Decision-makers must consider trade-offs between the various costs and benefits of different group testing technologies, including how many individuals to include per test, how many stages of testing to conduct, and other potential logistical challenges [63].

We predicted that silent introductions of SARS-CoV-2 led to large outbreaks in the absence of specific control strategies. This is consistent with large COVID-19 outbreaks observed in LTCFs worldwide [6, 8, 10, 16], including an infamous outbreak in early 2020 in King County, Washington that resulted in 167 confirmed infections within 3 weeks of the first reported case [5]. We further predicted that larger proportions of patients became infected than staff, consistent with emerging evidence of higher SARS-CoV-2 incidence in patients than staff across LTCF settings worldwide [3, 8]. We also predicted larger and more rapid outbreaks when SARS-CoV-2 was introduced through admission of an infected patient, rather than through a member of staff infected in the community, with important implications for surveillance efficacy (Additional File 2: Fig. S11). These findings are likely due to the nature of human interactions in the LTCF upon which we based our model, in which patient-patient contacts were particularly long and numerous [41]. Overall, these findings reinforce both (i) a need to screen incoming patients potentially exposed to or infected with SARS-CoV-2 [72] and (ii) the importance of interventions to limit contact between patients (e.g. social distancing among retirement home residents), as already widely recommended for affected facilities in the current pandemic context [4].

Simulated outbreaks were further characterized by delays between silent introduction of SARS-CoV-2 and first onset of COVID-19 symptoms, during which time new infections not (yet) showing symptoms accumulated. This is consistent with reported transmission dynamics of SARS-CoV-2; for instance, modelling studies have estimated that 30–57% of secondary infections among identified transmission pairs resulted from pre-symptomatic transmission [49], and that, early on in COVID-19 outbreaks in Singapore and Tianjin, pre-symptomatic transmission accounted for at least 65% of all transmission events [19]. Findings are also consistent with high proportions of asymptomatic infection, and important roles for pre-symptomatic and asymptomatic transmission reported in various LTCF outbreaks [5, 16, 21, 26–29]. The often silent nature of SARS-CoV-2 transmission highlights epidemiological challenges associated with screening for emerging outbreaks using symptoms alone. In addition to the strategies highlighted above, we found that testing patients and healthcare workers with any and not only severe COVID-like symptoms can substantially improve outbreak detection, supporting recommendations to expand testing criteria in LTCFs to include individuals with atypical signs and symptoms of COVID-19, such as muscle aches, sore throat, and chest pain [72].

A strength of the present study is the use of highly detailed inter-individual contact data to inform our individual-based transmission model. This allowed for recreation of life-like interaction dynamics among and between LTCF patients and staff and facilitated simulation of more realistic SARS-CoV-2 dissemination than a traditional mass-action transmission process. We are aware of no other studies using detailed individual-level contact networks to simulate SARS-CoV-2 transmission in healthcare settings, nor of studies using transmission modelling to evaluate COVID-19 surveillance strategies for emerging outbreaks.

Previous studies of COVID-19 surveillance have largely focused on the ability of testing strategies to mitigate ongoing SARS-CoV-2 transmission in active outbreak settings [73]. In particular, contact tracing has been identified as a highly effective form of surveillance, by targeting testing and isolation interventions to individuals at high risk of infection [74, 75]. However, these findings have limited relevance for emerging outbreaks, where active SARS-CoV-2 infection and ongoing transmission are not yet known. For healthcare facilities vulnerable to SARS-CoV-2 introductions, specific surveillance strategies are required for initial outbreak detection, in order to alert healthcare professionals and decision-makers to the presence of the virus in their institutions. Only then can proven measures like contact tracing and case isolation be implemented. By targeting this important epidemiological context, our findings complement an existing evidence base that has until now largely focused on how to control outbreaks that are already detected and well underway.

This work has several limitations. First, substantial uncertainties remain regarding epidemiological characteristics of COVID-19. It is well established that various COVID-19 outcomes vary with age, comorbidity, and frailty [76–78], but quantitative descriptions of these associations are incomplete, and it was not possible to reliably integrate such individual-level variation into our model. For instance, owing to individual-level risk factors, higher rates of symptomatic infection may be expected among LTCF residents than staff. Yet an outbreak investigation across six London care homes experiencing COVID-19 outbreaks estimated similar rates of asymptomatic infection in patients and staff and found no association with age [79], while the meta-analysis used to inform asymptomatic infection in our work highlighted poor reporting of age in included studies, precluding quantification of its relationship to COVID-19 symptom risk [51]. Nonetheless, calibrating model parameters to individual-level risk factors would facilitate more realistic simulations, and accounting for potentially higher rates of severe infection among older and frailer individuals could result in improved performance of symptom-based surveillance, including corresponding cascades and group testing strategies. This distinction may be particularly relevant for hospices, nursing homes, and other LTCFs with particularly frail populations; however, patients in the present rehabilitation hospital population were relatively young (median 58 years, IQR 47–72), limiting potential impacts of age-stratified disease progression in this study.

Other epidemiological uncertainties that we were unable to account for include temporal variability in SARS-CoV-2 transmissibility over the infectious period, individual-level variation in transmissibility, and a potential role for environmental acquisition [80, 81], although recent evidence suggests the former may be of limited relevance [82]. Further, most LTCFs have already implemented control measures, such as interruption of social activities and provisioning of personal protective equipment, that should act to reduce transmission from baseline. We conducted sensitivity analyses to consider unusually high and low transmission rates to reflect these uncertainties. Although SARS-CoV-2 spread more or less quickly, the relative efficacies of surveillance strategies were largely unchanged in these analyses, resulting in the same conclusions for optimizing use of limited testing resources to detect COVID-19 outbreaks (Additional File 2: Figs. S9, S10, S12, S13).

Second, LTCFs represent a diverse range of healthcare institutions, each with unique specializations, patient populations and living conditions, and the generalizability of our findings across these settings is not clear. In a sensitivity analysis, we restricted simulations to the 30-bed geriatric ward to approximate a smaller LTCF geared towards elder care, with an average 8.0 daily patient-patient contacts and 8.3 daily patient-staff contacts. This compares to patterns observed in a nursing home in Paris (5.0 daily patient-patient contacts, 6.3 daily patient-staff contacts) [83], and corresponding results may better reflect a nursing home environment than the baseline analysis. In this much smaller facility, high testing and swabbing capacities approximated universal testing strategies, in which large proportions of individuals were routinely tested. This explains why randomly testing among all individuals was among the most effective strategies at highest testing capacity (Additional File 2: Fig. S8), and why pooling even relatively small numbers of randomly selected individuals was a particularly efficient strategy in this setting (Additional File 2: Fig. S14). Otherwise, overall conclusions for surveillance were similar to the baseline LTCF.

Finally, the testing landscape for COVID-19 is due to shift quickly, with increased testing capacity and alternative testing technologies, such as rapid diagnostic tests, likely to become increasingly available in the coming months and years. However, uptake of new technologies is certain to be heterogeneous, and testing resources may remain limited for the foreseeable future, particularly in low- and middle-income settings [31, 32]. To reflect a scenario with more effective testing technology, in a sensitivity analysis, we assumed higher and more stable RT-PCR sensitivity over the course of infection. In this analysis, qualitative surveillance conclusions were again unchanged from the main analysis, although testing patients upon LTCF admission was notably more effective than in the main analysis (Additional File 2: Fig. S15). Although we explicitly modelled standard RT-PCR testing throughout this study, our findings may be broadly generalizable to other COVID-19 testing technologies with limited capacity. Findings for group testing, however, necessarily assume that pooling clinical samples is both logistically feasible and retains sufficient diagnostic sensitivity, as demonstrated for RT-PCR and SARS-CoV-2. Further, even in settings with abundant testing capacity, limiting the number of tests necessary to detect an outbreak will remain a priority given health-economic concerns.

## Conclusions

In conclusion, our findings demonstrate the susceptibility of LTCFs to large COVID-19 outbreaks, as well as epidemiological challenges associated with COVID-19 surveillance. We found that testing cascades, which combine clinical indications and random testing, are a highly effective means to detect emerging outbreaks given ample testing resources. For resource-limited settings unable to routinely screen large numbers of individuals, however, group testing is preferable, both more effective and resource-efficient than cascades and other considered strategies. These findings add to a limited evidence base for optimizing COVID-19 surveillance in healthcare institutions. Even in regions where non-pharmaceutical interventions have come to slow transmission in the community, LTCFs remain uniquely vulnerable to COVID-19. Increasing testing capacity and expanding surveillance beyond symptom-based screening could allow for earlier outbreak detection, facilitating timely intervention to limit transmission and save lives.

## Supplementary information

**Additional file 1: Supplementary Methods.** (1) Data used to inform the model: **Table S1.** model transitions; **Table S2.** model parameters. (2) ODD protocol for individual-based modelling. (3) Description of the surveillance algorithm: **Figure S1.** RT-PCR sensitivity over time; **Figure S2.** RT-PCR sensitivity for pooled samples with a single positive specimen; **Figure S3.** RT-PCR sensitivity for pooled samples with multiple positive specimens.

**Additional file 2: Supplementary Results.** (1) LTCF demography: **Table S1.** Patient and staff population structure by ward. (2) COVID-19 outbreak characteristics: **Table S2.** cumulative outbreak size over time; **Table S3.** Time lags to first COVID-19 symptom onset; Table S4 – cumulative outbreak size upon first COVID-19 symptom onset; **Figure S1.** Simulated *R*_0_ distributions; **Figure S2.** comparing outbreak characteristics across five SARS-CoV-2 introduction scenarios. (3) Additional surveillance results: **Figure S3.** Relationship between detection lag and outbreak size upon detection; **Figure S4.** Probabilities of detecting outbreaks before nosocomial transmission and symptom onset; **Figure S5.** Comparing group testing efficacy in baseline and sensitivity analyses; **Figure S6.** Efficacy and resource use for selected surveillance strategies**; Figure S7.** incremental efficiency plots at high testing capacity; **Figure S8.** surveillance efficacy heatmaps for a 30-bed geriatric LTCF; **Figure S9.** surveillance efficacy heatmaps given a low SARS-CoV-2 transmission rate; **Figure S10.** surveillance efficacy heatmaps given a high SARS-CoV-2 transmission rate; **Figure S11.** the impacts of different routes of SARS-CoV-2 introduction and daily testing capacity on surveillance efficacy; **Figure S12.** incremental efficiency plots given a low SARS-CoV-2 transmission rate; **Figure S13.** incremental efficiency plots given a high SARS-CoV-2 transmission rate; **Figure S14.** incremental efficiency plots for a 30-bed geriatric LTCF; **Figure S15.** surveillance efficacy heatmaps given higher and more stable RT-PCR sensitivity.

## Data Availability

The datasets used and/or analysed during the current study are available from the corresponding author on reasonable request.

## Abbreviations

COVID-19: Coronavirus disease 2019
HCW: Healthcare worker
IER: Incremental efficiency ratio
LTCF: Long-term care facility
*R*_0_: Basic reproduction number
RT-PCR: Reverse transcriptase polymerase chain reaction
SARS-CoV-2: Severe acute respiratory syndrome coronavirus 2
SEIR: Susceptible Exposed Infectious Recovered
UK: United Kingdom
USA: United States of America
