# Supplementary material for: Optimizing COVID-19 surveillance in long-term care facilities: a modelling study
Source: BMC Med. 2020 Dec 8;18:386. doi: 10.1186/s12916-020-01866-6 (PMC7721547; doi:10.1186/s12916-020-01866-6)
Supplement: Supplementary file 1 — Additional file 1: Supplementary Methods. (1) Data used to inform the model: Table S1. model transitions; Table S2. model parameters. (2) ODD protocol for individual-based modelling. (3) Description of the surveillance algorithm: Figure S1. RT-PCR sensitivity over time; Figure S2. RT-PCR sensitivity for pooled samples with a single positive specimen; Figure S3. RT-PCR sensitivity for pooled samples with multiple positive specimens. [file 12916_2020_1866_MOESM1_ESM.docx]

**Additional File 1:** Supplementary methods for the article *Optimizing COVID-19 surveillance in long-term care: a modelling study*

David RM Smith*^1,2,3^, Audrey Duval*^1,2^, Koen B Pouwels^4,5^, Didier Guillemot^1,2,6^, Jérôme Fernandes^7^, Bich-Tram Huynh^1,2^, Laura Temime§^3,8^, Lulla Opatowski§^1,2^, on behalf of the AP-HP/Universities/Inserm COVID-19 research collaboration

1. Institut Pasteur, Epidemiology and Modelling of Antibiotic Evasion (EMAE), Paris, France
2. Université Paris-Saclay, UVSQ, Inserm, CESP, Anti-infective evasion and pharmacoepidemiology team, Montigny-Le-Bretonneux, France
3. Modélisation, épidémiologie et surveillance des risques sanitaires (MESuRS), Conservatoire national des arts et métiers, Paris, France
4. Health Economics Research Centre, Nuffield Department of Population Health, University of Oxford, Oxford, United Kingdom
5. The National Institute for Health Research (NIHR) Health Protection Research Unit in Healthcare Associated Infections and Antimicrobial Resistance, University of Oxford, Oxford, UK
6. AP-HP, Paris Saclay, Public Health, Medical Information, Clinical Research, Le Kremlin-Bicêtre, France
7. Clinique de soins de suite et réadaptation, Choisy-Le-Roi, France
8. PACRI unit, Institut Pasteur, Conservatoire national des arts et métiers, Paris, France

*contributed equally

§ contributed equally

**Introducing a SARS-CoV-2 transmission model for long-term care settings**

Nosocomial COVID-19 outbreaks were simulated using a dynamic, stochastic, individual-based model (IBM) coded in C++ with three main goals: (i) to use detailed inter-individual contact data to simulate dynamic contact networks among patients and staff in long-term care facility (LTCF) settings, (ii) to simulate transmission of SARS-CoV-2 among LTCF patients and staff in simulated contact networks, and (iii) to simulate clinical progression of COVID-19 among individuals infected with SARS-CoV-2 using a Susceptible Exposed Infectious Recovered (SEIR) process (throughout, the virus is referred to as SARS-CoV-2, and human infection with this virus as COVID-19). We subsequently developed a surveillance algorithm to evaluate different surveillance strategies (distribution of nasopharyngeal swabs and RT-PCR tests) for detection of simulated COVID-19 outbreaks.

First, we broadly introduce the data used to inform and parameterize the transmission model. Second, we describe the transmission model in full using the Overview, Design concepts, and Details (ODD) protocol for individual-based modelling. Third, we describe the surveillance algorithm and evaluation of different surveillance strategies.

1. **Data informing the individual-based model**

*Characterizing dynamic inter-individual contacts in an LTCF setting*

As described in the main text, LTCF structure, demographics, and dynamic contact networks were estimated using data from the i-Bird study. A statistical analysis of the i-Bird contact network has been published previously.[41] Briefly, using nearly 2.7 million close-proximity interactions (CPIs) recorded from 318 patients and 262 staff between July and October 2009, distinct contact patterns were identified for individuals in each ward, reflecting behaviours particular to patients and different types of staff in this LTCF. For instance, patients typically spent 24 hours per day in the facility and had higher rates of contact with HCWs during mornings and afternoons, but with other patients in evenings. Patients were potentially admitted or discharged over the course of the study period. The average patient length of stay was 7 weeks and a median 2 (range 0 - 11) new patients were admitted per day. Staff were present according to their respective working hours, and had fewer overall contacts during evenings and weekends. HCWs had more distinct contacts with other individuals (on average 14.3/day) than patients (11.2/day), but had a shorter cumulative duration of time spent in contact with others (15 minutes/day) than patients (32 minutes/day). Compared to other wards, contacts were fewest (8.6 distinct CPIs/day) and longest (47 cumulative minutes/day) in the geriatric ward. Further, in contrast to a contact network observed using similar methods in an acute care setting, patient-patient CPIs were particularly frequent and numerous in this LTCF.[41,42]

*Characterizing SARS-CoV-2 transmission along human contact networks*

In the transmission model, Susceptible patients and staff could become infected with COVID-19 if in direct contact with an Infectious individual. We assumed that the probability of transmission per infectious contact depends on the duration of that contact, *d* (limited to intervals of 30 seconds, the discrete time-step for the transmission model). This transmission probability was computed as follows. Assuming homogeneous mixing among individuals, the basic reproduction number (*R*_0_) of a pathogen can be approximated as

 (eq. 1)

where *p* is the per-minute probability of transmission between Susceptible and Infectious individuals in contact with one another; *n* is the average number of daily contacts per individual; *d* is the average duration of these contacts; and 𝜏 is the duration of the infectious period.[43] Using community estimates from France, including epidemiological modelling of COVID-19 transmission prior to lockdown (*R*_0_=3)[44] and a detailed survey of inter-individual contacts in the general community (*n* = 8 contacts/days, d = 30 minutes),[45] and assuming an infectious period of 𝜏 = 9 days for COVID-19, we calculated a transmission probability of *p* = 0.14% per minute spent in contact with a Susceptible individual. We further set a saturation threshold at one hour of contact, such that the per-contact transmission probability was at most 8.3% per contact between any two individuals. To reflect uncertainty in the transmissibility of COVID-19 per infectious contact, in sensitivity analyses we considered extreme estimates for COVID-19 epidemicity in the community (*R*_0_=1.5, 6), which translate to low and high transmission probabilities per minute spent in contact (*p*=0.07%, 0.28%).

*Characterizing COVID-19 infection*

The natural history of COVID-19 infection was conceptualized as a modified SEIR process. Corresponding with the model illustration in Figure 1C of the main text, the transitions of this model are described below in Table S1.

**Table S1.** State transitions for the SEIR infection process. Numerical values are drawn probabilistically for each transition for each individual in the IBM. Durations were taken as integer values.

| **Symbol** | **Name** | **Transition** | **Description** |
| --- | --- | --- | --- |
| *a* | Infection | S 🡪 E | After a contact with an Infectious individual, Susceptible individuals become infected and enter Exposed class with probability: (*p* × *duration of contact*) |
| *b* | Infectiousness onset (pre-smptomatic) | E 🡪 I_P_ | Exposed individuals become Infectious (but do not yet show symptoms) after duration *d_E_* |
| *c* | Sympton onset (severe) | I_P_ 🡪 I_S_ | Pre-symptomatic individuals progress to the next stage of infectiousness after duration *d_p_*; a proportion (*s_MS_*) × (*s_S_*) develop severe symptoms |
| *d* | Symptom onset (mild) | I_P_ 🡪 I_M_ | Pre-symptomatic individuals progress to the next stage of infectiousness after duration *d_p_*; a proportion (*s_MS_*) – (*s_MS_* × *s_S_*) develop mild symptoms |
| *e* | Symptom onset (asymptomatic) | I_P_ 🡪 I_A_ | Pre-symptomatic individuals progress to the next stage of infectiousness after duration *d_p,_;* a proportion (1 – *s_MS_*) are asymptomatic and never develop symptoms |
| *f* | Recovery | (I_S_ or I_M_ or I_A_) 🡪 R | Individuals progress to a Recovered state (non-infectious, non-symptomatic, non-susceptible to re-infection) after duration *d_S_* |

As described in the main text, clinical progression of COVID-19 was characterized by: (i) a non-infectious exposed period of 2-5 days, (ii) an infectious pre-symptomatic period of 1-3 days, (iii) an on-average 7-day infectious “symptomatic” period with three levels of symptom severity (severe, mild or asymptomatic), and (iv) eventual recovery with full immunity.

Similar infection processes have been used in contemporaneous COVID-19 modelling studies,[44,46-48], and sources for assumed parameter values are provided in parameter Table S2. Here, this structure allows for (i) a 3-8 day incubation period, (ii) 1 to 3 days of pre-symptomatic transmission, as well as (iii) potential asymptomatic transmission from individuals who never show symptoms.

**Table S2.** Model parameter estimates.

| **Parameter** | **Value (distribution)** | **Source** |
| --- | --- | --- |
| **Parameters for estimation of SARS-CoV-2 transmission rate per minute of contact** | | |
| SARS-CoV-2 basic reproduction number (*R*_0_) in the general community prior to lockdown | 3.0 (1.5, 6.0 in sensitivity analysis) | [44] |
| Average infectious period of SARS-CoV-2 (τ) | 9 days | [49] |
| Average number of contacts per day per individual prior to lockdown (*n*) | 8 | [45] |
| Average duration per contact prior to lockdown (*d*) | 30 minutes | [45] |
| **Epidemiological and clinical parameters** | | |
| SARS-CoV-2 transmission rate per minute of contact (*p*) | 0.00139 (0.00070, 0.00278 in sensitivity analysis) | Estimated |
| Duration of exposed period (latency) (*d_E_*) | 2-5 days (uniform) | Approximated from [18, 50] |
| Duration of pre-symptomatic period (*d_P_*) | 1-3 days (uniform) | Approximated from [18, 50] |
| Duration of symptomatic period  (whether asymptomatic, mild symptomatic or severe symptomatic) (*d_S_*) | 7 days (log-normal, σ² = 7) | Approximated from [49] |
| Proportion of COVID-19 infections presenting any symptoms (*s_MS_*) | 0.7 | [51] |
| Proportion of symptomatic COVID-19 infections with severe symptoms (*s_S_*) | 0.2 | [52] |
| **Testing and surveillance parameters** | | |
| Daily incidence proportion of non-COVID but COVID-like symptoms | 0.011 | Estimated from OSCOUR data, described in [53] |
| Proportion of non-COVID but COVID-like symptoms with signs of severity | 0.2 | Assumed |
| Delay from test to test result | 1 day | Assumed |
| RT-PCR specificity | 100% | [12] |
| RT-PCR sensitivity | Up to 80% (Figure S1) | [54] |
| Daily testing capacity (tests/day) | 1, 2, 4, 8, 16, 32 | Assumed |
| Maximum number of specimens per group test | 2, 4, 8, 16, 32, 64 | Assumed |
| RT-PCR sensitivity discounting rate per additional true-negative specimen | 0.7% (1.3% in sensitivity analysis) | Estimated from [38] |

1. **Description of the model using the ODD protocol**

The simulation model used in this study is CTCModeler, an individual-based model (IBM) developed to simulate pathogen transmission among and between patients and staff in healthcare settings.[39] The IBM was programmed in C++ with the repast HPC library 2.2.0. In the following, we describe the IBM as it was used for the present study, following the ODD protocol of Grimm et al.[40]

1. ***Purpose.***

The IBM simulates nosocomial transmission of SARS-CoV-2 between humans using inter-individual contact data. The main goals are (i) to simulate inter-individual contacts between individuals in an LTCF, (ii) to trace SARS-CoV-2 transmission along this contact network, and (iii) to describe clinical progression of COVID-19 infection among individuals infected with SARS-CoV-2.

1. ***Entities, State Variables and Scales.***

*Entities and state variables*

All entities in the model are conceived as existing within the LTCF. There are two main classes in the model: Individual and Pathogen. These are derived from the abstract Organism class, which is composed of common variables inherited by both Individual and Pathogen. The Individual class has two children, patient and staff (visitors were excluded). Pathogen has only one child, the virus SARS-CoV-2. Each object has a unique ID variable in each simulation.

Common variables for Individuals are anonymous hospital ID number, age, gender, type of individual (patient or staff), admission date, discharge date, allocated ward, a map of infection, current positive SAR-CoV-2 infection, and infection status (statuses following a modified SEIR process are described above). For patients, admission refers to arriving to the LTCF as a new patient, and discharge refers to leaving the LTCF.

The map of pathogen status contains the current status of infection as well as its duration, i.e. the date at which an individual passes from one infection state to the next. The patient class also includes a hospital flag variable, which describes the reason for hospitalization, while the staff class also includes a category variable, which describes that individual’s occupation. There are 13 occupations in total: caregiver, nurse, physiotherapist, occupational therapist, nurse trainee, physician, hospital porter, hospital services, administration, other rehabilitation staff, management, logistical staff, and activity coordinator/hairdresser.

The common variable for Pathogen is current positive individuals, which lists all individuals who are infected with the virus at a given point in time.

*Scales*

The model runs using discrete 30-second time-steps and each simulation is run for approximately 12 weeks (85 days).

There is no explicit spatial scale in the model; instead, individuals enter into contact with one another according to hourly probabilities of contact, stratified by type of individual, occupation (for staff), ward, time of day and day of the week (weekday vs. weekend). Simulation of this contact network is detailed further below.

1. ***Process Overview and Scheduling.***

Simulation processes are run on three time schedules: weeks, days and time steps.

Weeks. Each week, a spontaneous infection can occur either in a new patient upon their admission to the LTCF, or among admitted staff. The latter is conceptualized as infection occurring in the community (i.e. outside the LTCF). This scheduling process is only relevant for epidemiological scenarios with weekly introductions of SARS-CoV-2 (scenarios 1, 3, and 5). See the Details (initialization) section for more information on epidemiological scenarios considered.

Days. The model performs three actions on Individuals each day: change infection status, add individual (admission), or remove individual (discharge). Infection progression follows an SEIR process, described above and in the main text. Durations for each stage of infection for each individual are drawn probabilistically from their respective distributions (parameter values in Table S2). Individuals are added or removed based on daily admissions and discharges listed in the admission file from the i-Bird study. Readmissions are possible for both patients and staff. When individuals “leave” the simulation, they go into a transitory container that keeps track of their information (e.g. immunization status) in case later re-admitted.

Time Step. The model time step is 30 seconds. At each time step, the model simulates (i) contacts and (ii) possible SARS-CoV-2 transmission events between individuals, which are calculated using the sum total of the number of time steps in each contact.

1. ***Design concepts***

*Basic principles.*

This IBM uses detailed inter-individual contact data from the i-Bird study to build a stochastic, dynamic contact network between all Individuals in the LTCF over the 85-day simulation period. Individuals with a ‘Susceptible’ infection status (S) can become infected with SARS-CoV-2 if in contact with an Individual with an ‘Infectious’ infection status (I_P_, I_A_, I_M_, I_S_). This infection process is based on classical concepts from the mathematical modelling of infectious diseases. Both of these processes are conceptualized as ‘Interactions’ and are described below.

*Interactions.*

Two types of interactions occur during the simulation:

Inter-individual Contacts.

Patient and staff populations of the Berck-sur-Mer rehabilitation hospital in Northern France are used in the model. This includes 318 patients and 262 staff included in the i-Bird study and present in the LTCF over an 85-day period, described elsewhere.[41] Patients and staff are distributed across five wards, including three neurology wards, one nutrition ward and one geriatrics ward. 90 members of staff are not affiliated with any one particular ward and are instead grouped as being in the ‘Other’ ward. This includes both staff working in back offices (i.e. not on any particular ward), as well as staff who regularly work across multiple wards.

Real staff scheduling and patient admission data are used to determine who is ‘admitted’ in the model (i.e. present in the LTCF) on any given day of the 12-week simulation period. However, instead of using the raw contact measured in the i-Bird study, a novel contact network is simulated to account for missing data resulting from imperfect sensor compliance in the raw network.

For the simulated network, contact probabilities are estimated using hourly contact rates from the raw network for each type of individual, ward, time of day, and day of the week (weekday vs. weekend). Contact durations are drawn from log-normal distributions; duration means and standard deviations are also estimated from the raw network and stratified by these same variables.

At each time step, the model builds an edge between pairs of Individuals if newly in contact with one another (based on contact probabilities described above) and maintains the edge if still in contact (based on contact durations described above).

In a previous analysis, the simulated network was found to have a greater total number of contacts than the raw network, as expected to account for missing data, but contact durations were nearly identical and other network properties were similar; for instance, the degree of the patient-to-staff subgraph for the simulated network (8.98, 8.85 – 9.12) is comparable to the raw network (8.71, 8.52 – 8.91).[55]

Inter-individual Transmission. Infectious Individuals can transmit their Pathogen to Susceptible Individuals through the contact network. For each contact, the probability of transmission per minute spent in contact (*p*) rate is multiplied by the duration of the contact in minutes (*d*). Here, the model also incorporates transmission probability saturation at 60 minutes (such that *p* <= 8.3%). The infection process has no bearing on the infection status of the Individual acting as the Pathogen donor.

*Stochasticity.*

For each model scenario, CTCModeler is run 100 times to produce 100 distinct epidemic simulations (see details of different model scenarios below). This is done to account for stochasticity in three model process: (i) initialization conditions, (ii) COVID-19 life history, and (iii) SARS-CoV-2 transmission. (Additional stochasticity resulting from the surveillance algorithm is external to the transmission model and is described later.)

For (i), the first Individual infected with SARS-CoV-2 at t=0 (the index case) is randomly selected among patients newly admitted that same day, or among staff presently admitted (depending on the epidemiological scenario considered; see below). For scenarios with weekly SARS-CoV-2 introductions, infected patients and staff are always randomly selected in this way.

For (ii), durations of each stage of infection are drawn randomly from their respective probability distributions and rounded to the nearest integer value as soon as an Individual becomes infected.

For (iii), when a pair of Infectious and Susceptible Individuals are in contact with one another, a transmission event occurs if the calculated probability of transmission (*p* $\times$ *d*) is greater than or equal to a number randomly drawn from a uniform distribution bounded by 0% and 100%.

Although the contact network was generated stochastically as described above, the same contact network was used for each epidemic simulation, and hence variation between epidemic simulations does not result from stochasticity in inter-individual contacts.

**Details**

*Initialization*.

This section describes initialization conditions for running of the model, as well as the various scenarios and sensitivity analyses considered to account for model uncertainty.

Before the first time-step in each simulation, one randomly selected Individual is infected with non-symptomatic SARS-CoV-2 infection (with equal probabilities of their infection stage being exposed, infectious pre-symptomatic, or infectious asymptomatic). This first infection is referred to as the ‘index case’. Index cases are conceived as being either infected patients newly admitted to the LTCF upon transfer from another setting, or staff who acquired infection in the community.

The following describes how initialization conditions are adjusted in the model to allow simulation of various scenarios: (i) five distinct scenarios of SARS-CoV-2 introduction into the LTCF, (ii) the baseline 170-bed LTCF vs. a smaller 30-bed LTCF geared towards elder care, and (iii) three distinct transmission rates.

Epidemiological scenarios of SARS-CoV-2 introduction

Five distinct epidemiological scenarios are considered, each describing a different source and frequency of SARS-CoV-2 introduction(s) into the LTCF: (baseline scenario 1, weekly patient or staff) either one infected patient admitted or one staff member infected once weekly, assuming 50% probability of patient or staff each week; (scenario 2, single patient transfer) one infected patient admitted at *t*_0_; (scenario 3, weekly patient transfer), a different infected patient admitted once weekly; (scenario 4, single infected staff), one staff member infected in the community at *t*_0_; and lastly (scenario 5, weekly infected staff) a different staff member infected in the community once weekly.

Two distinct LTCFs

Two distinct LTCFs are considered. The baseline LTCF uses all Individuals in the LTCF from the i-Bird study as described above. The second LTCF excludes all Individuals from Wards 1, 2, 3 and 4, leaving only individuals in Ward 5 and the Other ward. For this second LTCF, a distinct contact network was simulated in the same way as described above for the baseline LTCF. All of the five epidemiological scenarios above were also run for this second LTCF.

SARS-CoV-2 transmission rate

To account for uncertainty in SARS-CoV-2 transmissibility, the baseline model (five-ward LTCF, introduction scenario 1) is also run using the extreme transmission rate estimates described above (*p*=0.07%, *p*=0.28%).

*Input.*

Three input files are needed to run the IBM (admission, contact and parameter files). The admission file lists dates of hospital arrival and departure for all individuals included in a simulation. The contact file lists all contacts that occur between individuals (patients and staff) over time during simulations. Here, simulated contact files were obtained as described above. The parameter file gathers all parameter values that the IBM needs.

A separate file containing parameter values was used as input to define all parameter values for Individuals. Parameter values were derived from the original contact network from the i-Bird study, including patient demographic information. However, parameters related to infection were not included, because there were simulated and modified over the course of simulations (described above).

1. **Description of the COVID-19 surveillance algorithm**

*Overview*

A surveillance algorithm was developed to distribute simulated nasopharyngeal swabs and RT-PCR tests to Individuals from the individual-based transmission model described above, and to test them for active SARS-CoV-2 infection. Swabs and tests were distributed once per day using clinical and demographic indicators and assuming a daily limit (capacity) to the number of swabs and tests available. The 14 surveillance strategies considered differ according to which individuals were selected each day for testing. These strategies are outlined further in the main text, and more information for group testing is provided below.

*Surveillance indications*

Included indications for surveillance were: LTCF admission (for patients only), type of individual (patient, HCW or ancillary staff), any COVID-like symptoms and severe COVID-like symptoms. Output files from epidemic simulations contained a table of all Individuals in the model, their unique ID number and their identity (patient or type of staff). For each day of each simulation, LTCF admission status and infection status for each Individual were also included. Surveillance indicators were obtained from these output files, and were supplemented with additional symptom data as described below.

*Running the surveillance algorithm*

Starting on the first day of each epidemic simulation (t=0), the surveillance algorithm was run 100 times to (i) identify which individuals were to be swabbed and tested, (ii) stochastically determine test results, and (iii) count the cumulative number of swabs and tests used. Individuals were selected randomly among those indicated if on a given day there were more Individuals indicated for testing than there were tests available. The algorithm continued until the first positive test result was returned, to a maximum of 22 days, after which all outbreaks were assumed to be detected. The strategies determining which individuals were indicated for testing are outlined in Table 1 in the main text. Each test result was determined using a stochastic process, with diagnostic sensitivity (the probability of a positive test result for a true SARS-CoV-2-positive specimen) depending on the duration of time since infection on the day that the corresponding swab was taken (detailed further below).

*Accounting for non-COVID but COVID-like symptoms*

We generated 100 ‘symptom incidence files’ for each epidemic simulation (1 for each run of the surveillance algorithm). Symptom incidence files indicate the first day that COVID-like symptoms emerged for each Individual, and their severity (mild or severe). Note that many Individuals never experienced COVID-19 symptoms.

Since actual COVID-19 symptoms were taken from output files from epidemic simulations, they were identical across all 100 symptom incidence files within each epidemic simulation. The daily incidence of non-COVID but COVID-like symptoms (approximately 1.1% of Individuals, see Table S2) was then used to randomly assign additional COVID-like symptoms (mild or severe) to randomly selected Individuals on each day. These non-COVID but COVID-like symptoms thus varied across each of the 100 symptom incidence files, which in the context of epidemiological surveillance were assumed to be indistinguishable from real COVID-19 symptoms.

*Defining group testing*

Group testing (sample pooling) is a form of surveillance in which clinical samples from multiple subjects are combined into a group sample and evaluated using a single diagnostic test. If the test result is positive, then at least one of the individual samples included in the group sample is also positive (excluding external factors such as contamination), indicating presence of active infection in the sample population. Provided it retains sufficient diagnostic sensitivity, group testing reduces the number of tests necessary to screen low-prevalence populations for pathogens.

In the present study, we evaluated classic two-stage Dorfman pooling, in which each individual included in a group sample is individually re-swabbed and re-tested if the group test result is positive. For simulation, we grouped clinical specimens from multiple nasopharyngeal swabs, and applied a single RT-PCR test to the group sample, again assuming a 1-day lag to a test result. We varied the maximum number of swabs included per test, reaching up to 2, 4, 8, 16, 32 or 64. Group test sensitivity was defined with an equation accounting for the sensitivity (true positive probability) for each individual swab included in the sample. Although positive tests resulted in re-swabbing and re-testing, for purposes of surveillance, outbreaks were considered to be detected upon the first positive test result (after the first round of testing) and not after case identification (second round).

*Diagnostic sensitivity of RT-PCR*

RT-PCR sensitivity varies over the course of infection, depending on the density of virus in the clinical sample. In a meta-analysis, the false negative rate (FNR) for RT-PCR detection of SARS-CoV-2 in nasopharyngeal samples was estimated as a function of time since infection.[54] On the first and second days after infection, this was 100%, falling to 67% by four days, reaching a minimum of 20% by eight days, and increasing gradually thereafter. Estimates were made until 21 days after infection; we extrapolated the curve linearly to reach 100% FNR at 28 days. In our algorithm, when an Individual infected with SARS-CoV-2 was selected for testing, the test result was determined by a stochastic process. Sensitivity on day *t* of that Individual’s infection (*s_t_*) was calculated as 1-FNR_t_ (Figure S1).

For individuals infected within the LTCF, duration of infection at each swab was calculated directly from outbreak simulations. However, it was necessary to estimate infection durations for individuals that entered the LTCF already infected (i.e. patients infected upon LTCF admission or staff infected in the community). Individuals entering in the E class were assumed to have acquired SARS-CoV-2 the previous day. For individuals entering in the I_P_ or I_A_ classes, duration of infection upon LTCF entry (and hence corresponding RT-PCR sensitivity) was calculated by randomly drawing durations from the duration distributions defined for preceding infection classes (see Tables S1 and S2).

In a sensitivity analysis, we considered higher and more stable RT-PCR sensitivity. Instead of varying by time since infection, in this analysis sensitivity was fixed at 30% during the exposed stage of infection (E), and 90% across all infectious stages (I_P_, I_A_, I_M_, I_S_).


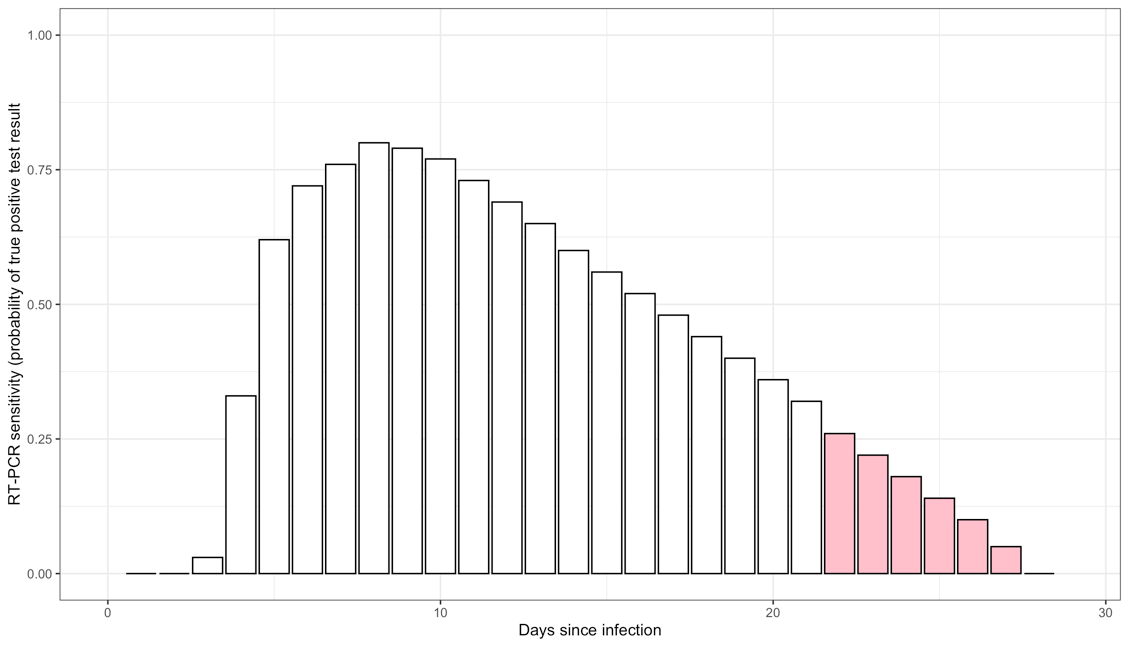


**Figure S1.** RT-PCR test sensitivity as a function of time since infection. Values were calculated from the RT-PCR false negative rate for detection of SARS-CoV-2 using upper respiratory samples, as estimated for up to 21 days in a meta-analysis by Kucirka et al.[54] Pink bars were extrapolated linearly from the data to complete the distribution.

*Estimating diagnostic sensitivity for group samples*

Diagnostic sensitivity of RT-PCR is necessarily reduced when a pathogen-positive sample is diluted with negative samples and/or less concentrated positive samples. For our algorithm, it was necessary to derive a formula describing RT-PCR sensitivity as a function of the sensitivity of each individual sample included in each group sample.

In an experimental study, Yelin *et al*. combined individual SARS-CoV-2-positive samples with between 1 and 63 negative samples, and evaluated the Cycle threshold (Ct) at which RT-PCR detected SARS-CoV-2 in each group sample (fluorescence threshold=300).[38] To define RT-PCR test results from these data, we defined a default diagnostic threshold for the main analysis (positive SARS-CoV-2 test result if fluorescence >300 at Ct=40) and a stricter threshold for sensitivity analysis (Ct=35). Using these criteria, we calculated the proportion of true positive test results (diagnostic sensitivity) for each sample size S (the total number of samples included per group sample), and defined group test sensitivity (*s_g_*) as a function of the number of negative samples included in the group sample (S-1). We assumed a linear relationship

 (eq. 2)

where *r* is the discounting rate per additional negative sample added to the group sample. In this experimental context, the intercept *s_0_* is fixed at 1 since the positive sample is a known positive. We estimated the discounting rate for both diagnostic cut-offs, finding *r*=0.690% at Ct=40 and *r*=1.271% at Ct=35 (Figure S2). These findings are broadly consistent with empirical data. In a large Spanish study evaluating 3,519 nasopharyngeal samples in ten-sample pools, group test sensitivity was estimated at 97.1% when accounting for only major discrepancies between individual and group test results, and 85.5% when accounting for both major and minor discrepancies.[56]


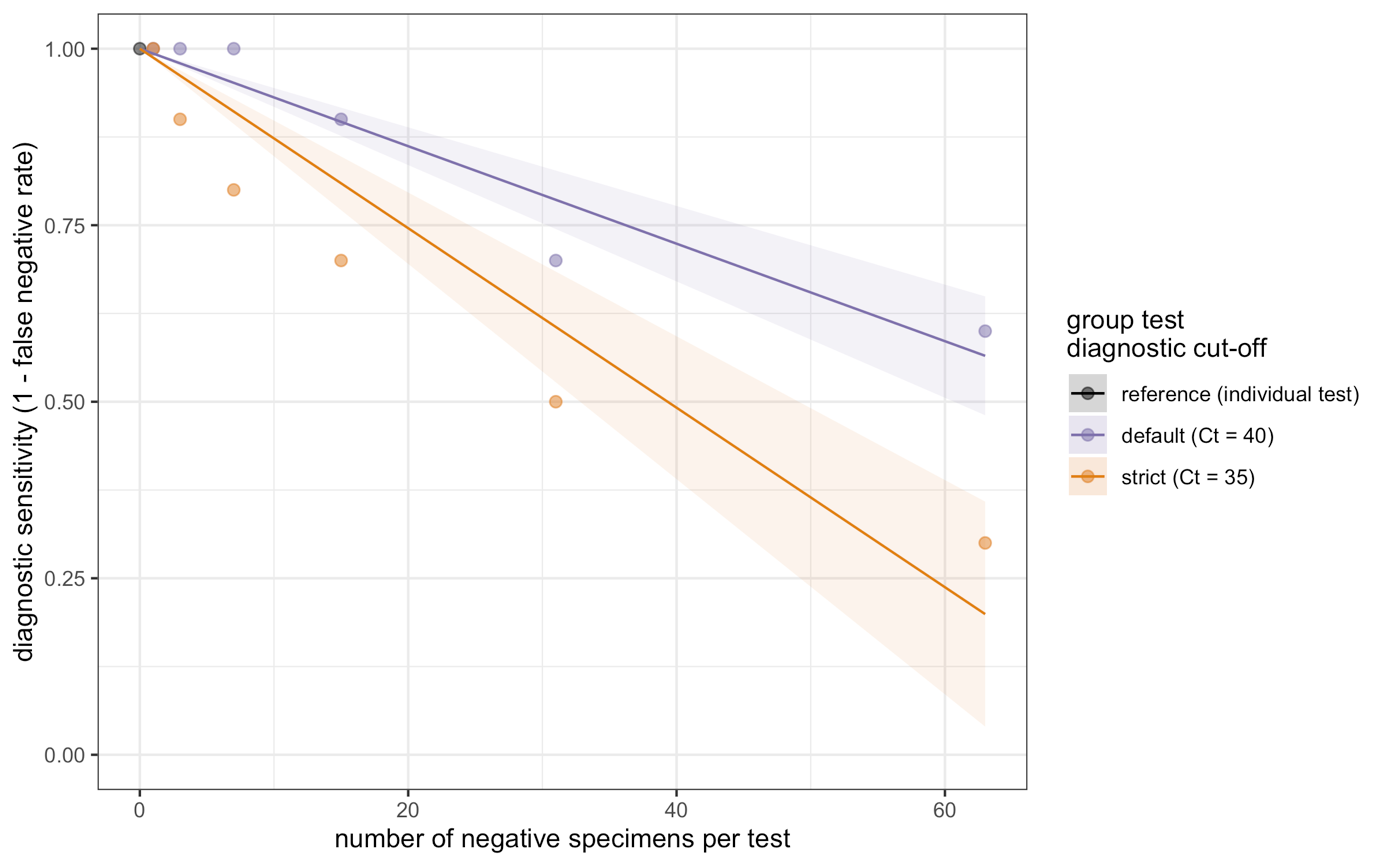


**Figure S2.** Diagnostic sensitivity of RT-PCR for detecting SARS-CoV-2 in a group sample declines as additional negative specimens are added to the group sample. Using data from Yelin et al. (circles), we used linear regression (lines) to estimate *r*, the discounting rate per additional negative specimen added to the sample. We found *r*=0.006904 for the diagnostic cut-off used in the baseline analysis (Ct=40) and *r*=0.01271 for the stricter cut-off used in sensitivity analysis (Ct=35). Here, there is only one individual SARS-CoV-2-positive sample included per group sample, regardless of the number of negative samples included.

*Accounting for multiple positive specimens in a group sample*

Methods have been proposed for using detailed virological data to calculate the diagnostic sensitivity for a sample containing multiple positive specimens.[57] In absence of such data, it was necessary to estimate diagnostic sensitivity for multi-positive group tests given only the number of specimens included, and the diagnostic sensitivity of each positive specimen when tested individually.

For a group sample consisting of multiple potentially detectable positive samples *P* and a set total number of samples *S*, the density of viral particles is necessarily less than the most concentrated sample *P_max_*, but more than the density given by a group sample with the same *S* but containing only *P_max_* and no other *P*. In this sense, the theoretical sensitivity of RT-PCR for a multi-positive group sample is bounded by *s_max_* (sensitivity for *P_max_* when tested individually) and *s_S-1_* (sensitivity for *P_max_* when diluted with S-1 negative samples, i.e. with no other *P*). By extension, when RT-PCR sensitivity is known for each individual sample *s_i_*, we propose that sensitivity for a group test can be approximated by using equation 2 to estimate sensitivity for *P_max_* alone, and then modifying this by the relative individual sensitivities *s_i_* of all other *P* included in the sample. This is given by

 (eq. 3)

where the relative contribution of each *P* (excluding *P_1_*, which gives *s_max_*) to *s_g_* is

. (eq. 4)

We demonstrate this equation below using seven hypothetical examples of group samples, each composed of *S* individual samples, of which *P* are potentially detectable SARS-CoV-2-positive samples. Figure S3 shows *s_i_* (triangles, the diagnostic sensitivity for each *P* when tested individually) and *s_g_* (circles, corresponding diagnostic sensitivity for the group test). The figure also illustrates the theoretical range of *s_g_* (grey shaded area) as a function of *S* for a given *s_max_* and *r*.


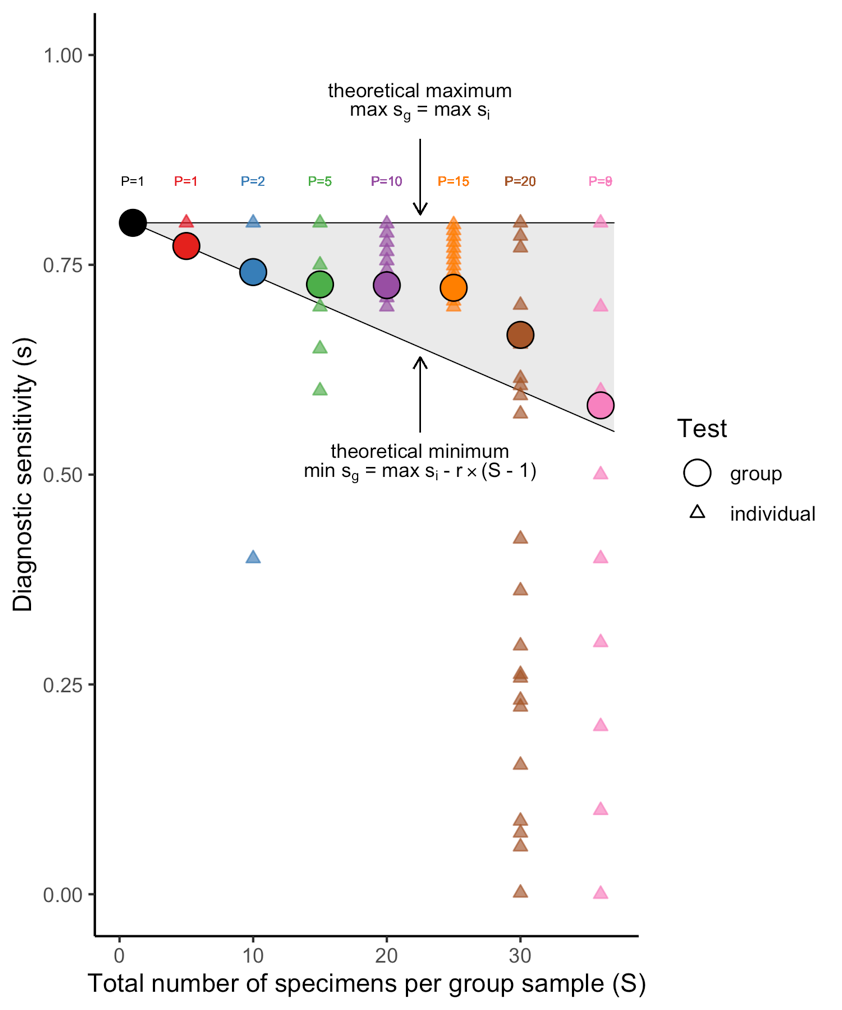


**Figure S3.** RT-PCR diagnostic sensitivity for a mixed group sample (*s_g_*, circles) was modelled as a function of sensitivity for each individual specimen in that sample (*s_i_*, triangles). Each colour corresponds to a different group. Only positive samples P are shown; negative and undetectable individual samples (*s_i_* = 0) are excluded from the plot. The shaded grey area gives the full theoretical range of *s_g_* (here, given *s_max_*=0.7 and *r*=0.006904).
