# Supplementary material for: Optimizing COVID-19 surveillance in long-term care facilities: a modelling study
Source: BMC Med. 2020 Dec 8;18:386. doi: 10.1186/s12916-020-01866-6 (PMC7721547; doi:10.1186/s12916-020-01866-6)
Supplement: Supplementary file 2 — Additional file 2: Supplementary Results. (1) LTCF demography: Table S1. Patient and staff population structure by ward. (2) COVID-19 outbreak characteristics: Table S2. cumulative outbreak size over time; Table S3. Time lags to first COVID-19 symptom onset; Table S4 – cumulative outbreak size upon first COVID-19 symptom onset; Figure S1. Simulated R0 distributions; Figure S2. comparing outbreak characteristics across five SARS-CoV-2 introduction scenarios. (3) Additional surveillance results: Figure S3. Relationship between detection lag and outbreak size upon detection; Figure S4. Probabilities of detecting outbreaks before nosocomial transmission and symptom onset; Figure S5. Comparing group testing efficacy in baseline and sensitivity analyses; Figure S6. Efficacy and resource use for selected surveillance strategies; Figure S7. incremental efficiency plots at high testing capacity; Figure S8. surveillance efficacy heatmaps for a 30-bed geriatric LTCF; Figure S9. surveillance efficacy heatmaps given a low SARS-CoV-2 transmission rate; Figure S10. surveillance efficacy heatmaps given a high SARS-CoV-2 transmission rate; Figure S11. the impacts of different routes of SARS-CoV-2 introduction and daily testing capacity on surveillance efficacy; Figure S12. incremental efficiency plots given a low SARS-CoV-2 transmission rate; Figure S13. incremental efficiency plots given a high SARS-CoV-2 transmission rate; Figure S14. incremental efficiency plots for a 30-bed geriatric LTCF; Figure S15. surveillance efficacy heatmaps given higher and more stable RT-PCR sensitivity. [file 12916_2020_1866_MOESM2_ESM.docx]

**Additional File 2:** Supplementary results for the article *Optimizing COVID-19 surveillance in long-term care: a modelling study*

David RM Smith*^1,2,3^, Audrey Duval*^1,2^, Koen B Pouwels^4,5^, Didier Guillemot^1,2,6^, Jérôme Fernandes^7^, Bich-Tram Huynh^1,2^, Laura Temime§^3,8^, Lulla Opatowski§^1,2^, on behalf of the AP-HP/Universities/Inserm COVID-19 research collaboration

1. Institut Pasteur, Epidemiology and Modelling of Antibiotic Evasion (EMAE), Paris, France
2. Université Paris-Saclay, UVSQ, Inserm, CESP, Anti-infective evasion and pharmacoepidemiology team, Montigny-Le-Bretonneux, France
3. Modélisation, épidémiologie et surveillance des risques sanitaires (MESuRS), Conservatoire national des arts et métiers, Paris, France
4. Health Economics Research Centre, Nuffield Department of Population Health, University of Oxford, Oxford, United Kingdom
5. The National Institute for Health Research (NIHR) Health Protection Research Unit in Healthcare Associated Infections and Antimicrobial Resistance, University of Oxford, Oxford, UK
6. AP-HP, Paris Saclay, Public Health, Medical Information, Clinical Research, Le Kremlin-Bicêtre, France
7. Clinique de soins de suite et réadaptation, Choisy-Le-Roi, France
8. PACRI unit, Institut Pasteur, Conservatoire national des arts et métiers, Paris, France

*contributed equally

§ contributed equally

**Table S1.** Demographic breakdown of patients and staff present in each ward of the simulated baseline LTCF. Staff were grouped as healthcare workers (HCWs) or ancillary staff. The ward ‘Other’ accounts for staff not affiliated with any one particular ward, including those who work in the back office or regularly move between wards. In combination with Other staff, the geriatric ward (Ward 5, with its distinct patients, staff and within-ward contact network) was considered separately in a sensitivity analysis to simulate outbreaks in a smaller, 30-bed geriatric LTCF.

| **Type of individual** | | **Average number present per week per ward (% of all individuals present per ward)** | | | | | | |
| --- | --- | --- | --- | --- | --- | --- | --- | --- |
|  |  | **Ward 1** | **Ward 2** | **Ward 3** | **Ward 4** | **Ward 5** | **Other** | **All** |
| Patient | Patient | 38 (49%) | 32 (45%) | 35 (55%) | 35 (67%) | 30 (54%) | 0 (0%) | 170 (41%) |
| HCW | Caregiver | 20 (26%) | 19 (27%) | 14 (21%) | 7 (13%) | 10 (18%) | 21 (23%) | 92 (22%) |
|  | Nurse | 11 (14%) | 7 (10%) | 8 (13%) | 6 (11%) | 7 (13%) | 12 (13%) | 51 (12%) |
|  | Physiotherapist | 0 (0%) | 0 (0%) | 0 (0%) | 0 (0%) | 0 (0%) | 12 (13%) | 12 (3%) |
|  | Nurse trainee | 1 (1%) | 3 (5%) | 2 (3%) | 0 (0%) | 2 (3%) | 2 (2%) | 10 (2%) |
|  | Occupational Therapist | 0 (0%) | 0 (0%) | 0 (0%) | 0 (0%) | 0 (0%) | 7 (8%) | 7 (2%) |
|  | Physician | 1 (1%) | 2 (3%) | 1 (2%) | 1 (2%) | 1 (2%) | 0 (0%) | 6 (1%) |
|  | Hospital porter | 0 (0%) | 0 (0%) | 0 (0%) | 0 (0%) | 0 (0%) | 6 (6%) | 6 (1%) |
| Ancillary | Hospital services staff | 4 (5%) | 4 (6%) | 3 (5%) | 2 (4%) | 3 (5%) | 0 (0%) | 16 (4%) |
|  | Logistical staff | 0 (0%) | 0 (0%) | 0 (0%) | 0 (0%) | 0 (0%) | 16 (18%) | 16 (4%) |
|  | Other rehabilitation staff | 1 (1%) | 3 (4%) | 0 (0%) | 0 (0%) | 2 (4%) | 2 (2%) | 8 (2%) |
|  | Administrative staff | 0 (0%) | 0 (0%) | 0 (0%) | 0 (0%) | 0 (0%) | 8 (9%) | 8 (2%) |
|  | Management | 1 (1%) | 1 (1%) | 1 (2%) | 1 (2%) | 1 (2%) | 2 (2%) | 7 (2%) |
|  | Activity coordinator/ hairdresser | 0 (0%) | 0 (0%) | 0 (0%) | 0 (0%) | 0 (0%) | 2 (2%) | 2 (0%) |
| **Total (100%)** | | **78** | **72** | **63** | **53** | **55** | **90** | **410** |

**Table S2.** Simulated cumulative COVID-19 case counts over time, stratified by epidemiological scenario, LTCF, and type of individual. Only simulations resulting in outbreaks were included (for LTCF 1, 64% of simulations from scenario 4, 100% from other scenarios; for LTCF 2, 96% from scenario 2, 24% from scenario 4, 100% from other scenarios).

| **Scenario** | **Type of individual** | **Median cumulative number infected per outbreak by time *t* (95% UI)** | | | | | |
| --- | --- | --- | --- | --- | --- | --- | --- |
|  |  | **LTCF 1: Rehabilitation hospital (170 beds)** | | | **LTCF 2: Geriatric LTCF (30 beds)** | | |
|  |  | **1 week (t=7)** | **2 weeks (t=14)** | **3 weeks (t=21)** | **1 week (t=7)** | **2 weeks (t=14)** | **3 weeks (t=21)** |
| Scenario 1: Weekly patient or staff | Patient | 3 (0 - 16) | 21 (0 - 84) | 64 (2 - 142) | 1 (0 - 10) | 7 (0 - 27) | 24 (0 - 31) |
|  | HCW | 1 (0 - 5) | 5 (0 - 25) | 22 (1 - 77) | 1 (0 - 2) | 2 (0 - 12) | 8 (1 - 18) |
|  | Ancillary staff | 0 (0 - 1) | 1 (0 - 5) | 3 (0 - 13) | 0 (0 - 1) | 1 (0 - 3) | 2 (0 - 5) |
|  | **Total** | **4 (1 - 20)** | **30 (2 - 100)** | **86 (6 - 224)** | **2 (1 - 12)** | **10 (2 - 39)** | **34 (3 - 52)** |
| Scenario 2: Single patient transfer | Patient | 7 (2 - 19) | 30 (9 - 86) | 96 (33 - 145) | 3 (1 - 11) | 19 (1 - 26) | 27 (3 - 31) |
|  | HCW | 1 (0 - 5) | 8 (1 - 25) | 32 (7 - 68) | 0 (0 - 3) | 5 (0 - 11) | 12 (1 - 17) |
|  | Ancillary staff | 0 (0 - 2) | 1 (0 - 4) | 4 (0 - 11) | 0 (0 - 1) | 0 (0 - 2) | 2 (0 - 5) |
|  | **Total** | **9 (2 - 23)** | **40 (10 - 111)** | **133 (39 - 221)** | **4 (1 - 13)** | **25 (2 - 36)** | **40 (7 - 49)** |
| Scenario 3: Weekly patient transfer | Patient | 7 (2 - 19) | 37 (11 - 93) | 109 (43 - 148) | 4 (1 - 11) | 17 (2 - 26) | 28 (12 - 32) |
|  | HCW | 1 (0 - 4) | 9 (2 - 28) | 36 (11 - 76) | 0 (0 - 4) | 4 (0 - 12) | 11 (1 - 18) |
|  | Ancillary staff | 0 (0 - 2) | 1 (0 - 4) | 5 (0 - 12) | 0 (0 - 1) | 0 (0 - 3) | 2 (0 - 5) |
|  | **Total** | **9 (2 - 21)** | **48 (15 - 116)** | **150 (62 - 227)** | **4 (1 - 13)** | **23 (3 - 38)** | **42 (13 - 51)** |
| Scenario 4: Single infected staff | Patient | 0 (0 - 6) | 3 (0 - 41) | 25 (0 - 97) | 0 (0 - 4) | 4 (0 - 22) | 18 (0 - 29) |
|  | HCW | 1 (0 - 4) | 3 (1 - 14) | 8 (1 - 43) | 1 (0 - 2) | 2 (1 - 7) | 6 (2 - 13) |
|  | Ancillary staff | 0 (0 - 1) | 0 (0 - 3) | 1 (0 - 6) | 0 (0 - 1) | 0 (0 - 1) | 1 (0 - 3) |
|  | **Total** | **2 (1 - 9)** | **6 (2 - 57)** | **36 (2 - 146)** | **2 (1 - 6)** | **6 (2 - 29)** | **26 (2 - 44)** |
| Scenario 5: Weekly infected staff | Patient | 0 (0 - 11) | 2 (0 - 48) | 20 (0 - 108) | 0 (0 - 4) | 0 (0 - 19) | 0 (0 - 29) |
|  | HCW | 1 (0 - 4) | 3 (1 - 15) | 10 (1 - 40) | 1 (0 - 2) | 2 (0 - 7) | 3 (1 - 13) |
|  | Ancillary staff | 0 (0 - 1) | 1 (0 - 3) | 1 (0 - 6) | 0 (0 - 1) | 1 (0 - 2) | 1 (0 - 4) |
|  | **Total** | **2 (1 - 13)** | **6 (2 - 64)** | **34 (3 - 150)** | **1 (1 - 6)** | **2 (2 - 25)** | **4 (3 - 44)** |

**Table S3.** Lags between introduction of the index case and first presentation of mild and severe COVID-19 symptoms. Results are stratified by epidemiological scenario and LTCF. Only simulations resulting in outbreaks were included (for LTCF 1, 64% of simulations from scenario 4, 100% from other scenarios; for LTCF 2, 96% from scenario 2, 24% from scenario 4, 100% from other scenarios). Lags for outbreaks in which symptoms never appeared are indicated as ‘never’.

| **Scenario** | **Lag until first symptom onset, days (95% CI)** | | | |
| --- | --- | --- | --- | --- |
|  | **LTCF 1: Rehabilitation hospital** | | **LTCF 2: Nursing home** | |
|  | **Mild symptoms** | **Severe symptoms** | **Mild symptoms** | **Severe symptoms** |
| Scenario 1: Weekly patient or staff | 9 (2-24) | 15 (4-28) | 9 (2-28) | 18 (2 - never) |
| Scenario 2: Single patient transfer | 7 (2-15) | 12 (4-21) | 9 (2-24) | 15 (3 - never) |
| Scenario 3: Weekly patient transfer | 7 (2-13) | 10 (4-23) | 8 (2-19) | 14 (3 - never) |
| Scenario 4: Single infected staff | 8 (2-never) | 20 (3-never) | 17 (2-never) | 27 (3 - never) |
| Scenario 5: Weekly infected staff | 10 (2-37) | 21 (4-never) | 9 (2-28) | never (3 - never) |

**Table S4.** The cumulative number of individuals infected on the day that COVID-19 symptoms first appeared among anyone present in the LTCF (outbreak size upon first symptom onset). Results are stratified by epidemiological scenario and LTCF. Only simulations resulting in outbreaks were included (for LTCF 1, 64% of simulations from scenario 4, 100% from other scenarios; for LTCF 2, 96% from scenario 2, 24% from scenario 4, 100% from other scenarios), and outbreaks were excluded if no symptoms ever occurred over the course of the outbreak.

| **Scenario** | **Outbreak size upon first symptom onset (95% UI)** | | | |
| --- | --- | --- | --- | --- |
|  | **LTCF 1: Rehabilitation hospital** | | **LTCF 2: Nursing home** | |
|  | **Any symptoms** | **Severe symptoms** | **Any symptoms** | **Severe symptoms** |
| Scenario 1: Weekly patient or staff | 6 (1-30) | 26 (1-102) | 3 (1-24) | 24 (1-49) |
| Scenario 2: Single patient transfer | 8 (1-25) | 26 (3-100) | 7 (1-22) | 25 (3-42) |
| Scenario 3: Weekly patient transfer | 7 (1-24) | 23 (4-97) | 5 (1-19) | 24 (1-45) |
| Scenario 4: Single infected staff | 2 (1-20) | 18 (1-115) | 2 (1-9) | 22 (2-43) |
| Scenario 5: Weekly infected staff | 2 (1-23) | 26 (1-89) | 2 (1-20) | 20 (1-44) |

**Figure S1.** Distributions of the basic reproduction number (*R*_0_), the number of secondary infections caused by the initial index case introduced at the beginning of each simulation. In the baseline LTCF and under baseline assumptions (*p*=0.14%), *R*_0_ varied from 0 to 20 across 200 independent, stochastic simulations. The index case was a patient in 100 simulations (red) and a member of staff in the other 100 (blue). Overall, the mean value across all simulations was *R*_0_=4.04 (dashed black line), but *R*_0_ was greater when index cases were patients (mean *R*_0_=6.64) than staff (mean *R*_0_=1.44).

**
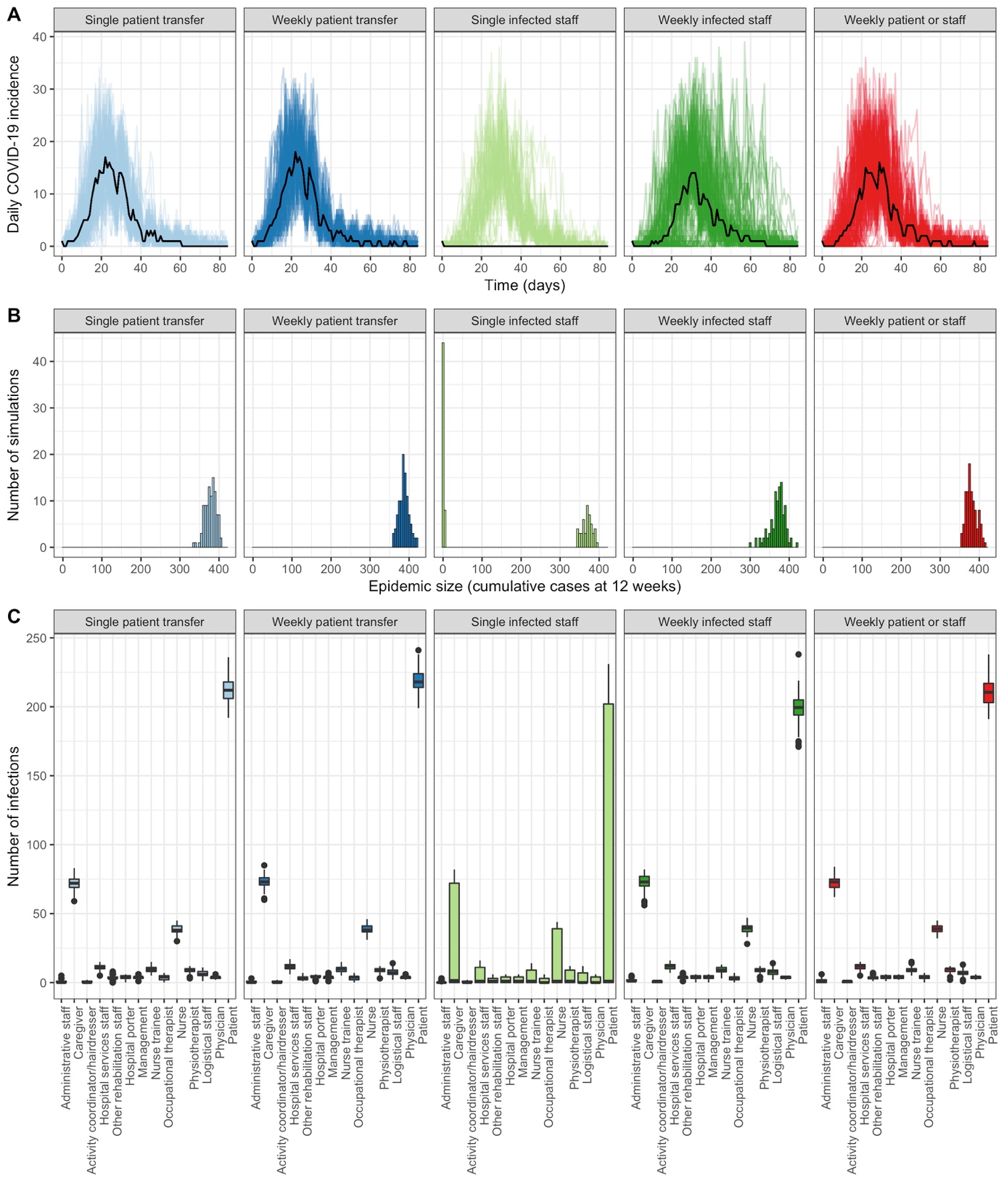
**

**Figure S2.** Epidemiological characteristics of COVID-19 epidemics simulated over a 12-week period in the baseline LTCF, in the absence of any surveillance, control measures or interventions, and comparing different scenarios of SARS-CoV-2 introduction into the LTCF (columns). **(A)** The daily incidence of COVID-19 infection among all patients and staff, with each coloured line representing a different simulation. Black lines represent the median daily incidence across all simulations. **(B)** Histograms of the final epidemic size at 12 weeks (NB: data are naturally censured by the 12-week simulation period). **(C)** Distributions of cumulative infection totals at 12 weeks among the fourteen different categories of individual present in the LTCF.

**
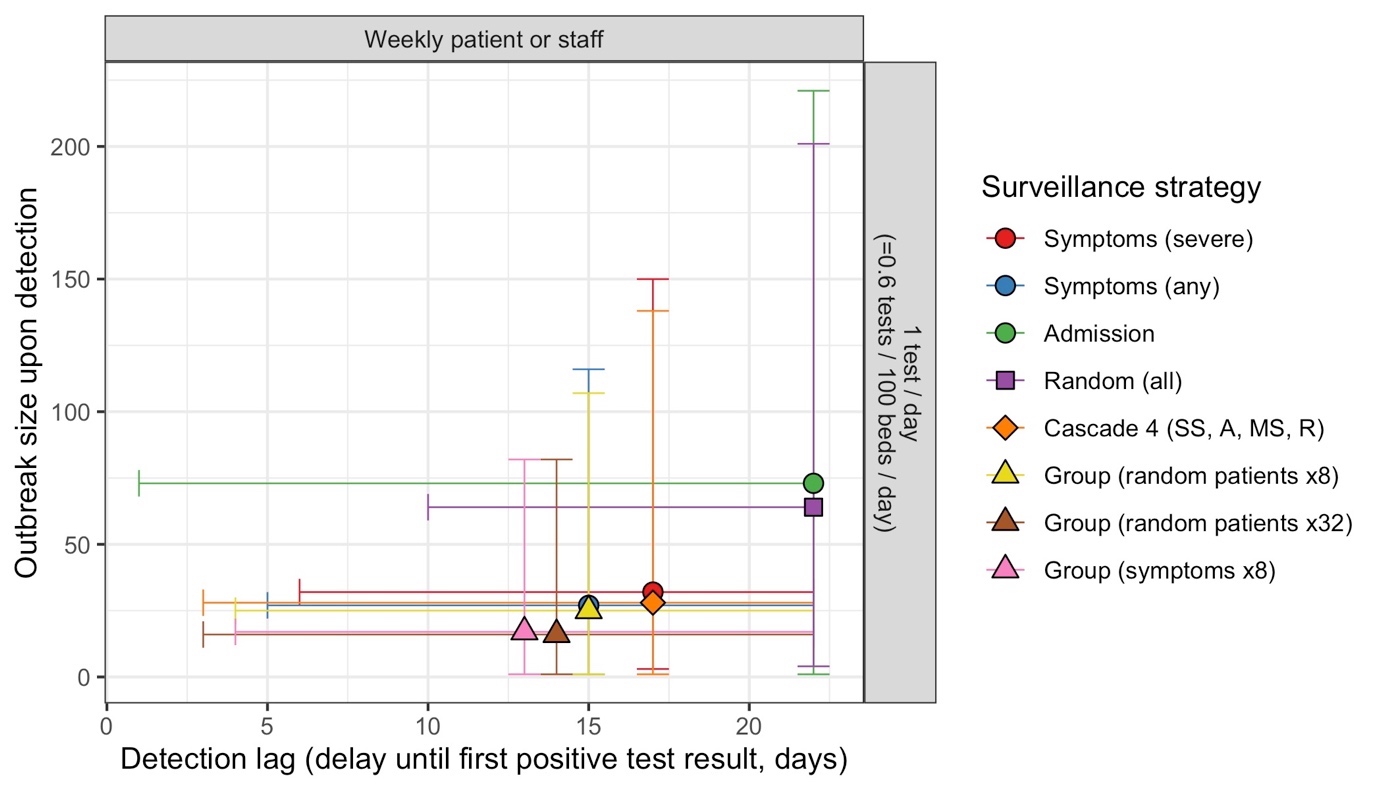
**

**Figure S3**.  Relationship between detection lag (x-axis) and the number of undetected cases upon outbreak detection (y-axis), for selected surveillance strategies (colours), for baseline assumptions and at a testing capacity of 1 test/day. Symbols represent medians and error bars represent 95% uncertainty intervals.

**
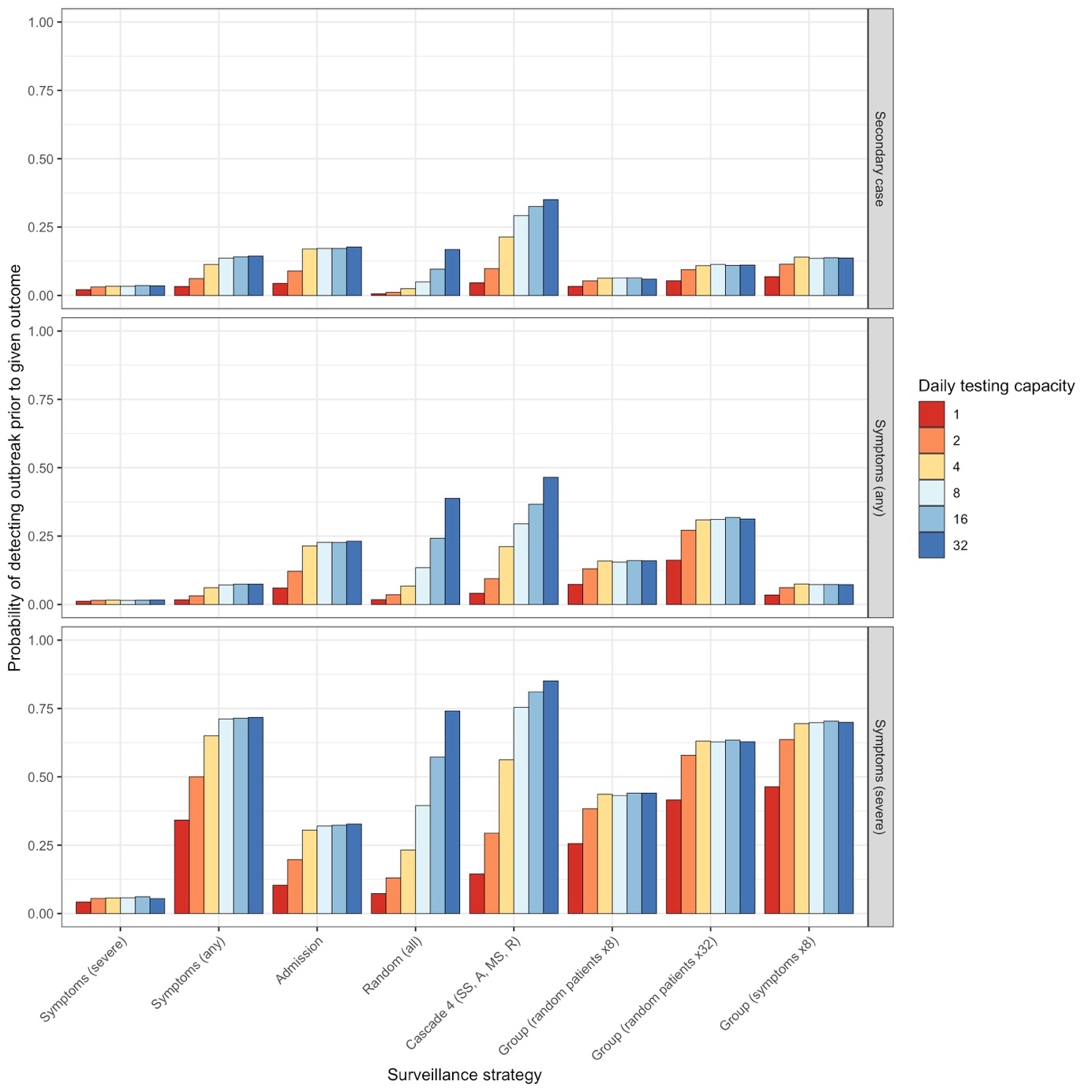
**

**Figure S4**. Probability of detecting COVID-19 outbreaks before any secondary cases (top panel), before the onset of any COVID-19 symptoms (middle panel), and before the onset of any severe COVID-19 symptoms (bottom panel) for baseline modelling assumptions. Probabilities depended on the surveillance strategy considered (x-axis) and the daily testing capacity (colours), and saturate at high testing capacity for all but random and cascade strategies.

**
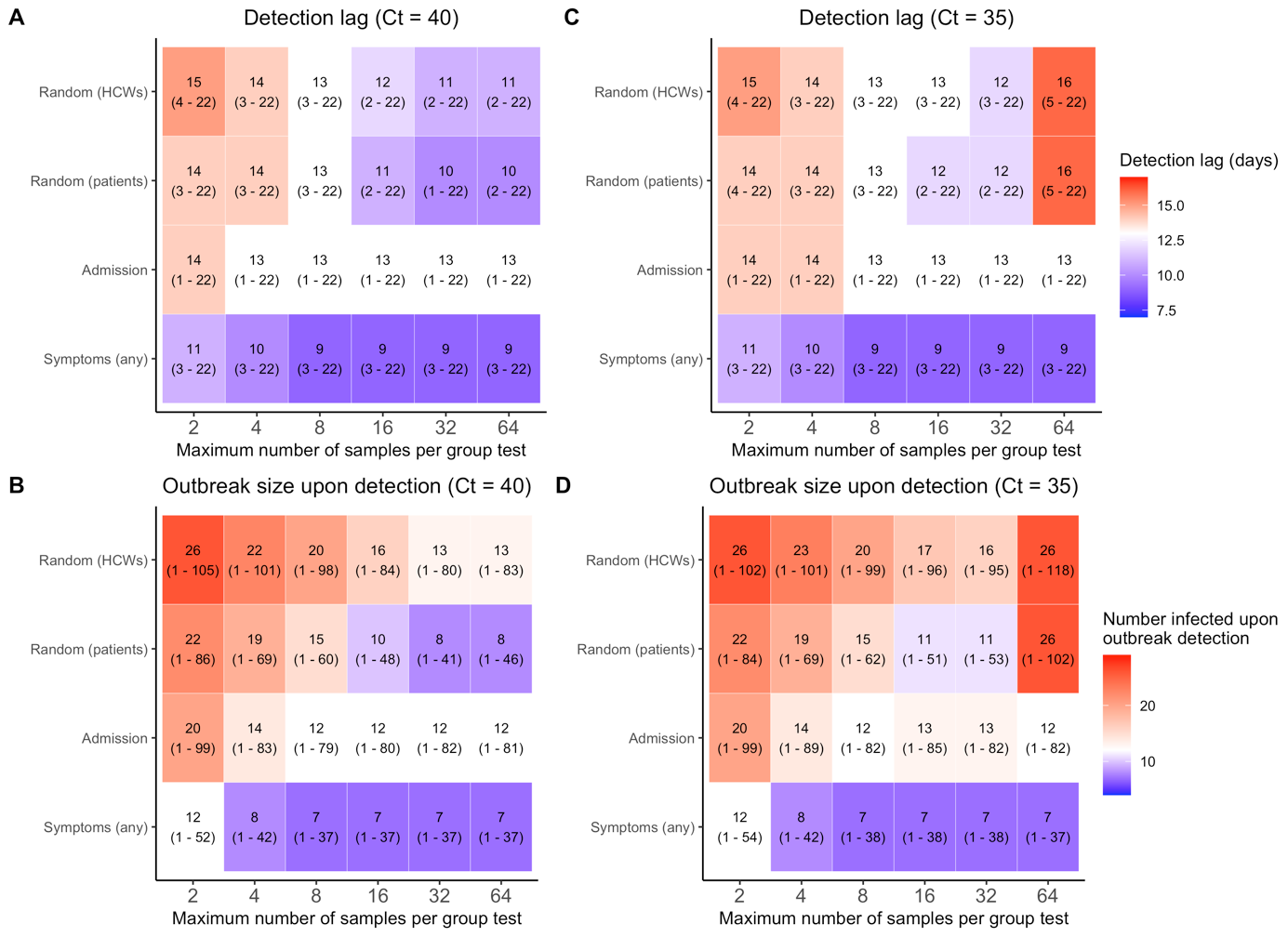
**

**Figure S5.** Group testing efficacy depends on which individuals are included in group samples (y-axis) and the maximum number of individual samples included per group sample (x-axis). Efficacy is visualized as a heat map where each tile corresponds to the median outcome (95% uncertainty intervals in parentheses) for two outcomes (detection lag, top panels; outbreak size upon detection, bottom panels) and for baseline modelling assumptions. Two Cycle thresholds (Ct) are considered, which correspond to diagnostic thresholds for RT-PCR testing. **(A)** Detection lag and **(B)** outbreak size upon detection for the baseline Cycle threshold value used in the main analysis (Ct=40). **(C)** Detection lag and **(D)** outbreak size upon detection for the stricter threshold used in sensitivity analysis.

**
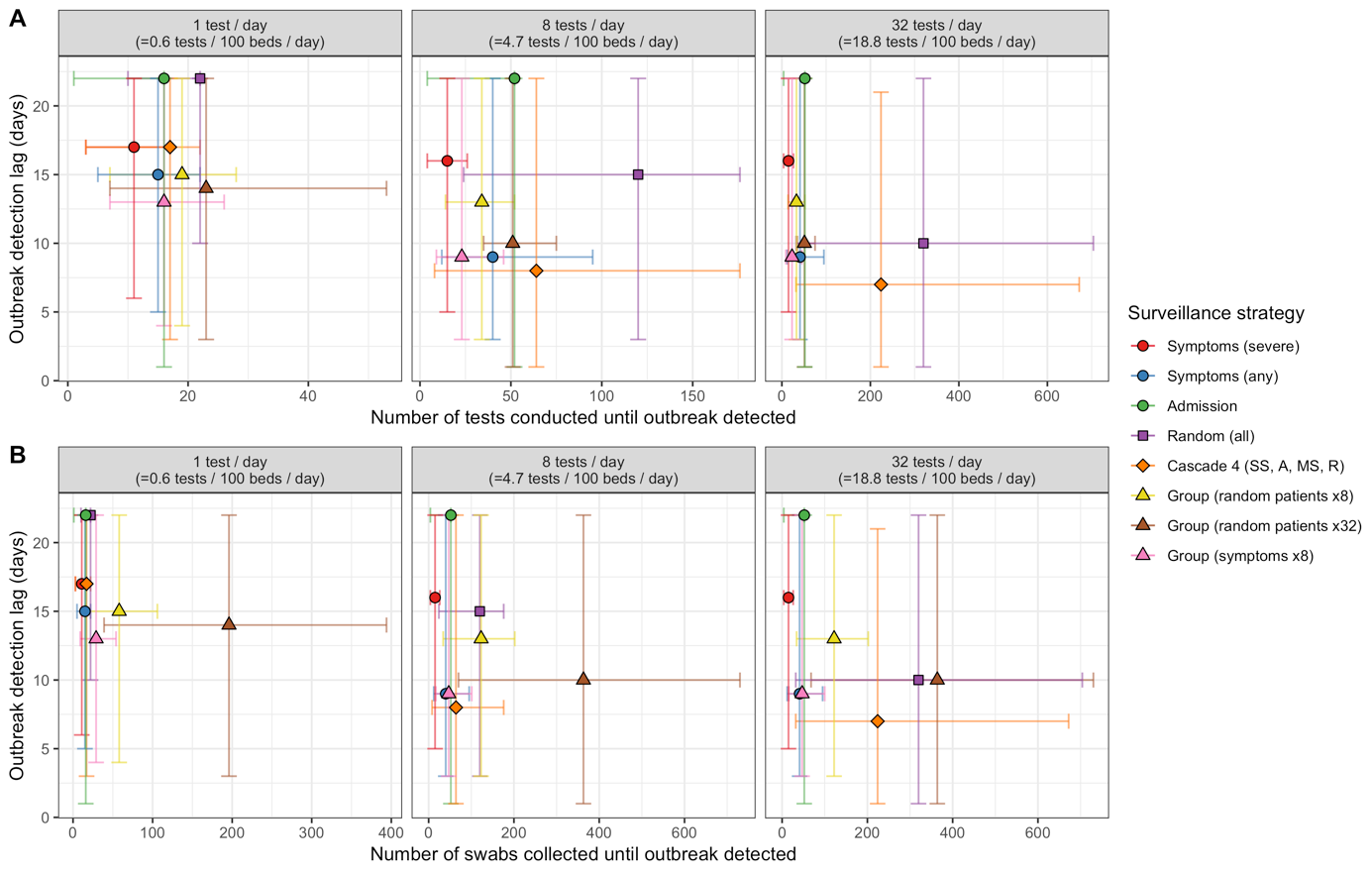
**

**Figure S6.** Efficiency plots for selected surveillance strategies given baseline modelling assumptions, comparing the efficacy (y-axis) and resource use (x-axis) in terms of the number of tests used (top row) and swabs collected (bottom row) until outbreaks were detected. The assumed daily testing capacity varies across columns. Symbols represent medians and error bars represent 95% uncertainty intervals across all outbreak simulations. For cascades: SS=severe symptoms, MS=mild symptoms, A=admission, R=random (patients).


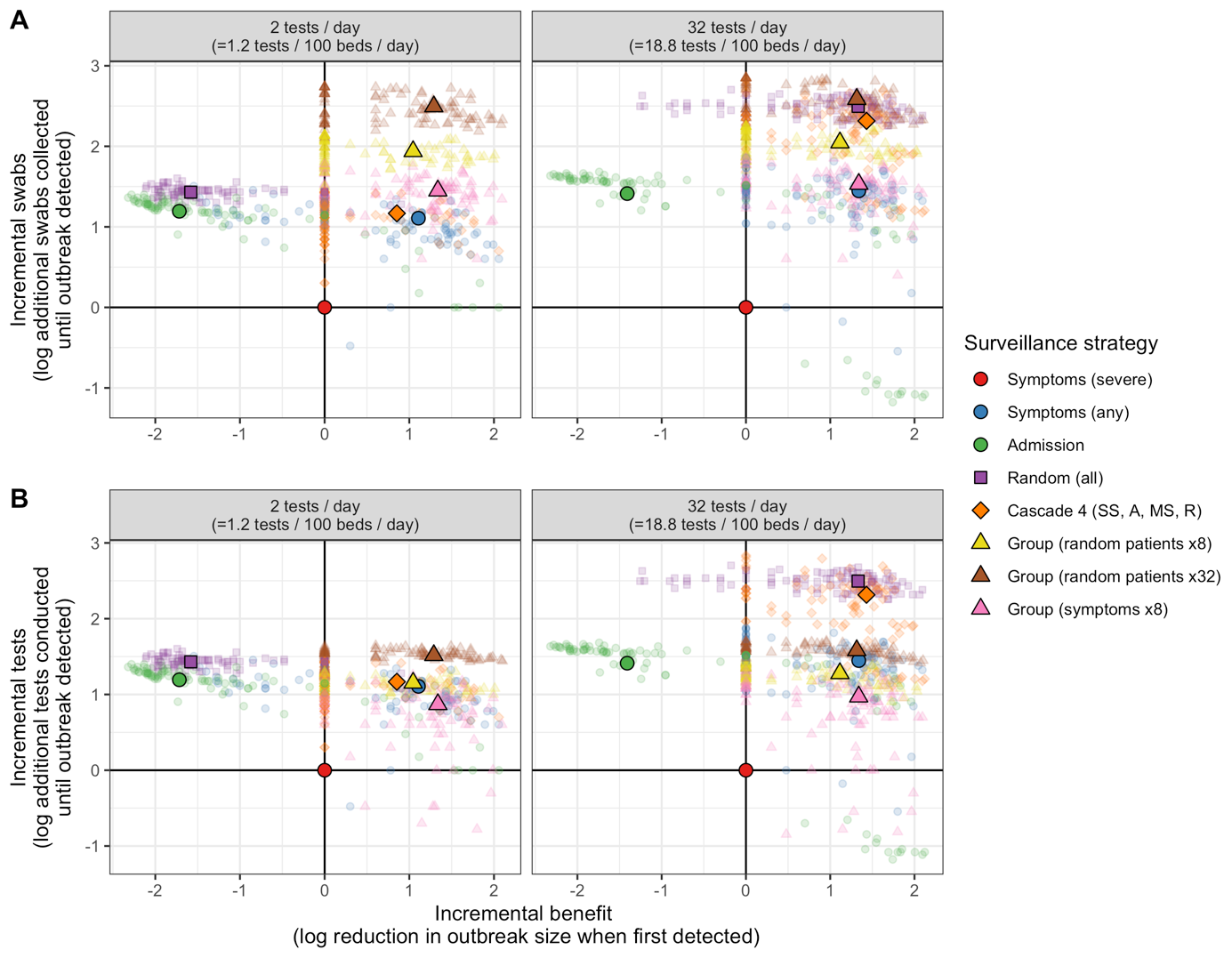


**Figure S7.** Log_10_-transformed incremental efficiency plots for simulations under baseline modelling assumptions, comparing incremental resource-use in terms of **(A)** nasopharyngeal swabs used and **(B)** RT-PCR tests conducted, and varying testing capacity from 2 tests/day (left panels) to 32 tests/day (right panels). Small translucent points represent median outcomes across 100 surveillance simulations for each simulated outbreak, and larger opaque points represent the mean of all medians.


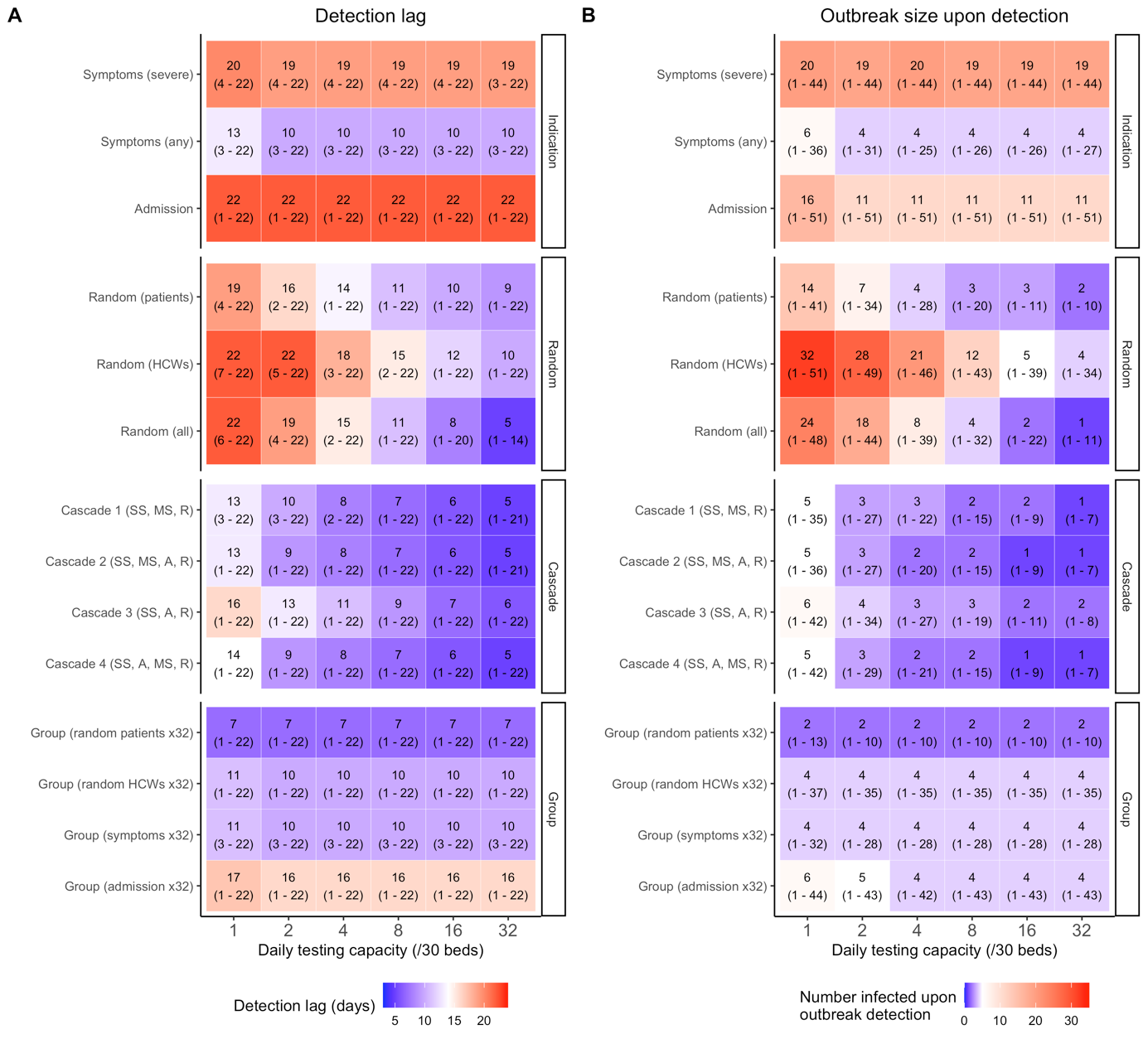


**Figure S8.** For simulations in the 30-bed geriatric LTCF, **(A)** median lags to outbreak detection (95% uncertainty interval) and **(B)** corresponding outbreak sizes upon detection are shown for each surveillance strategy (y-axis) as a function of the daily testing capacity (x-axis). Group testing strategies assume a maximum of 32 swabs per test.


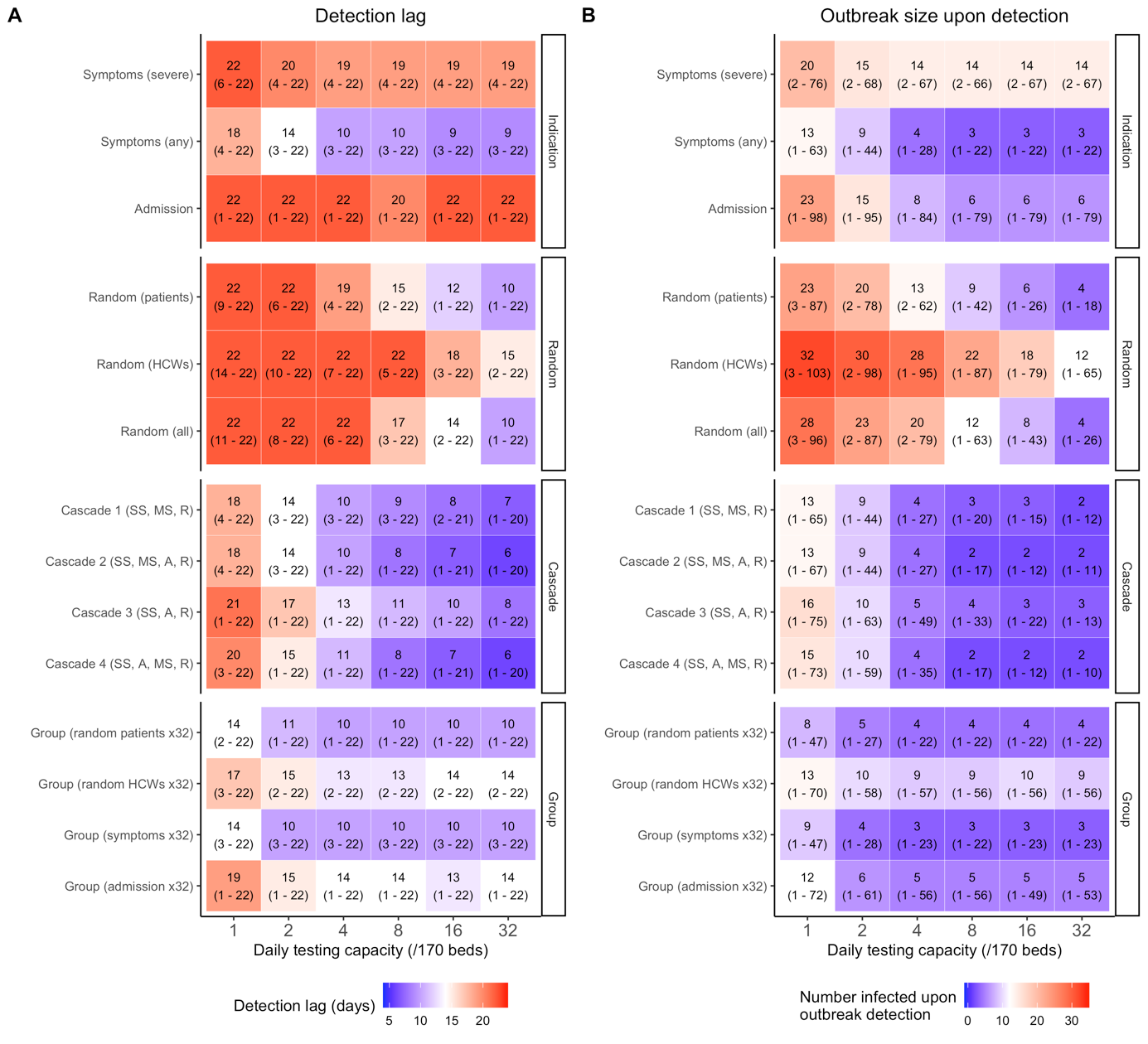


**Figure S9.** For simulations in the baseline LTCF with a low transmission rate (*p*=0.07%), **(A)** median lags to outbreak detection (95% uncertainty interval) and **(B)** corresponding outbreak sizes upon detection are shown for each surveillance strategy (y-axis) as a function of the daily testing capacity (x-axis). Group testing strategies assume a maximum of 32 swabs per test.


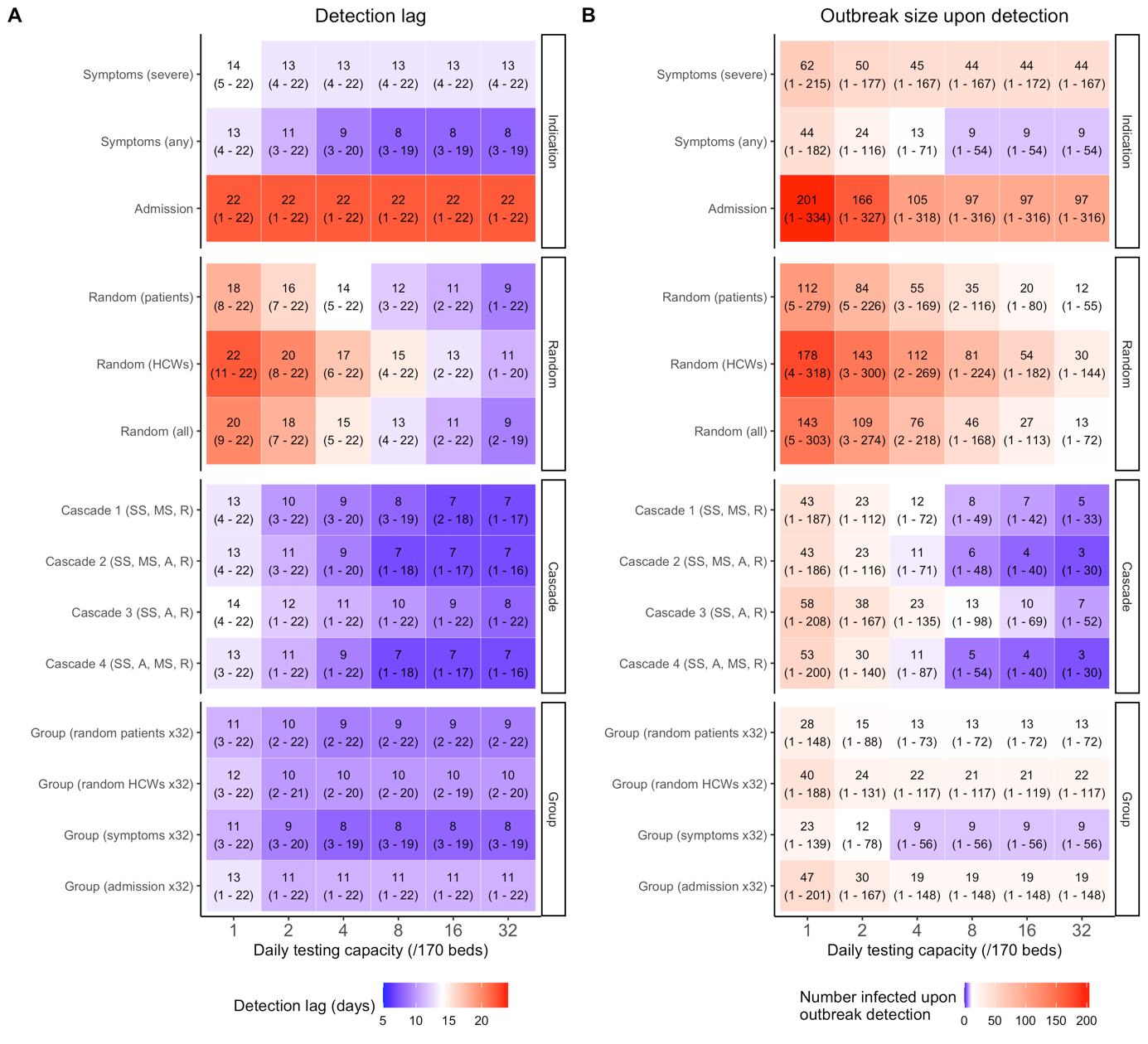


**Figure S10.** For simulations in the baseline LTCF with a high transmission rate (*p*=0.28%), **(A)** median lags to outbreak detection (95% uncertainty interval) and **(B)** corresponding outbreak sizes upon detection are shown for each surveillance strategy (y-axis) as a function of the daily testing capacity (x-axis). Group testing strategies assume a maximum of 32 swabs per test.

**
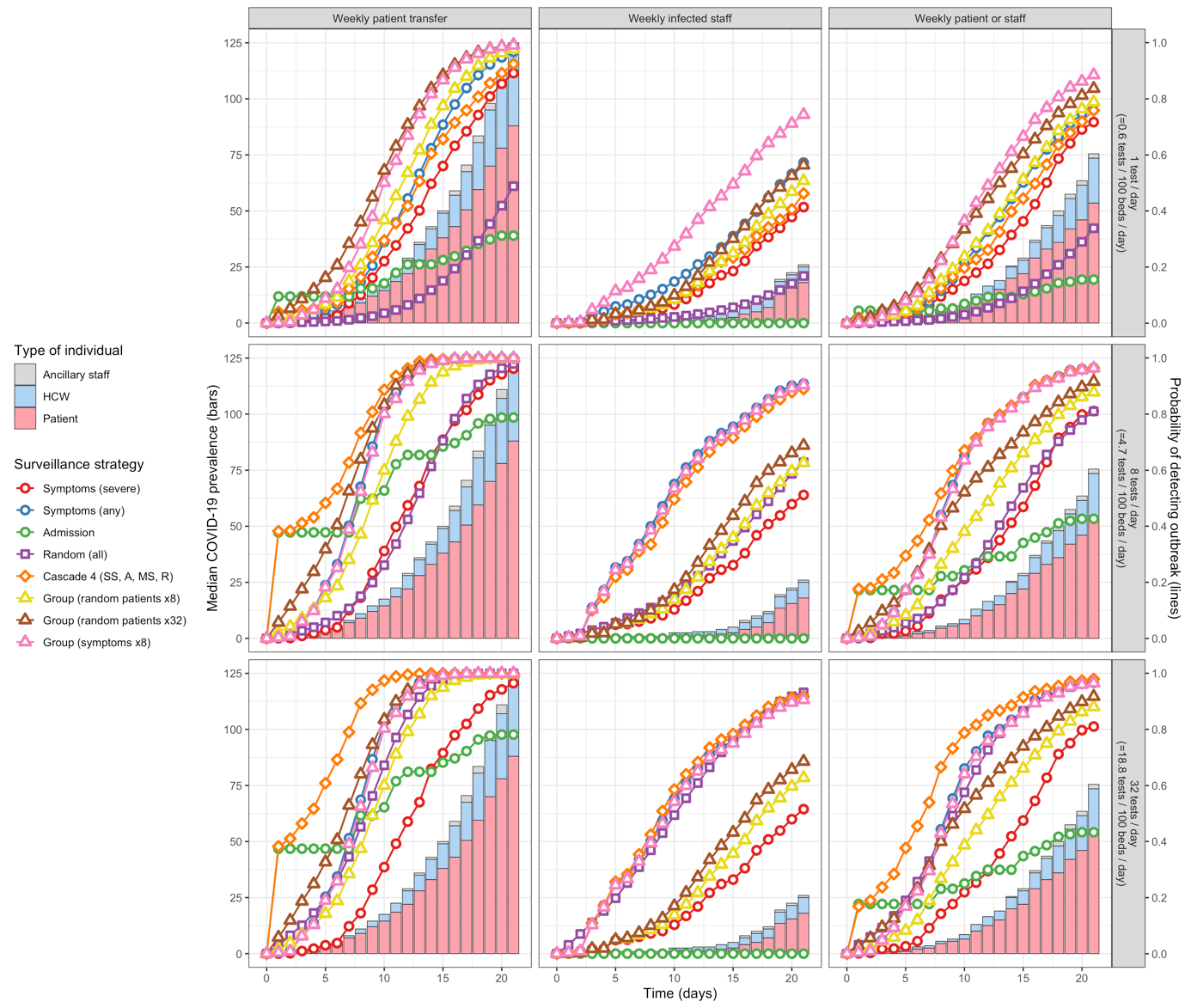
**

**Figure S11.** The size of COVID-19 outbreaks varied over time (coloured vertical bars) across SARS-CoV-2 importation scenarios (columns). Consequently, the probability of detecting outbreaks over time using different surveillance strategies (coloured lines) varied as a result of differences in how many, and which types of individuals became infected over time across these different importation scenarios; further, the efficacy of different surveillance strategies in these different scenarios depended on daily testing capacity (rows).


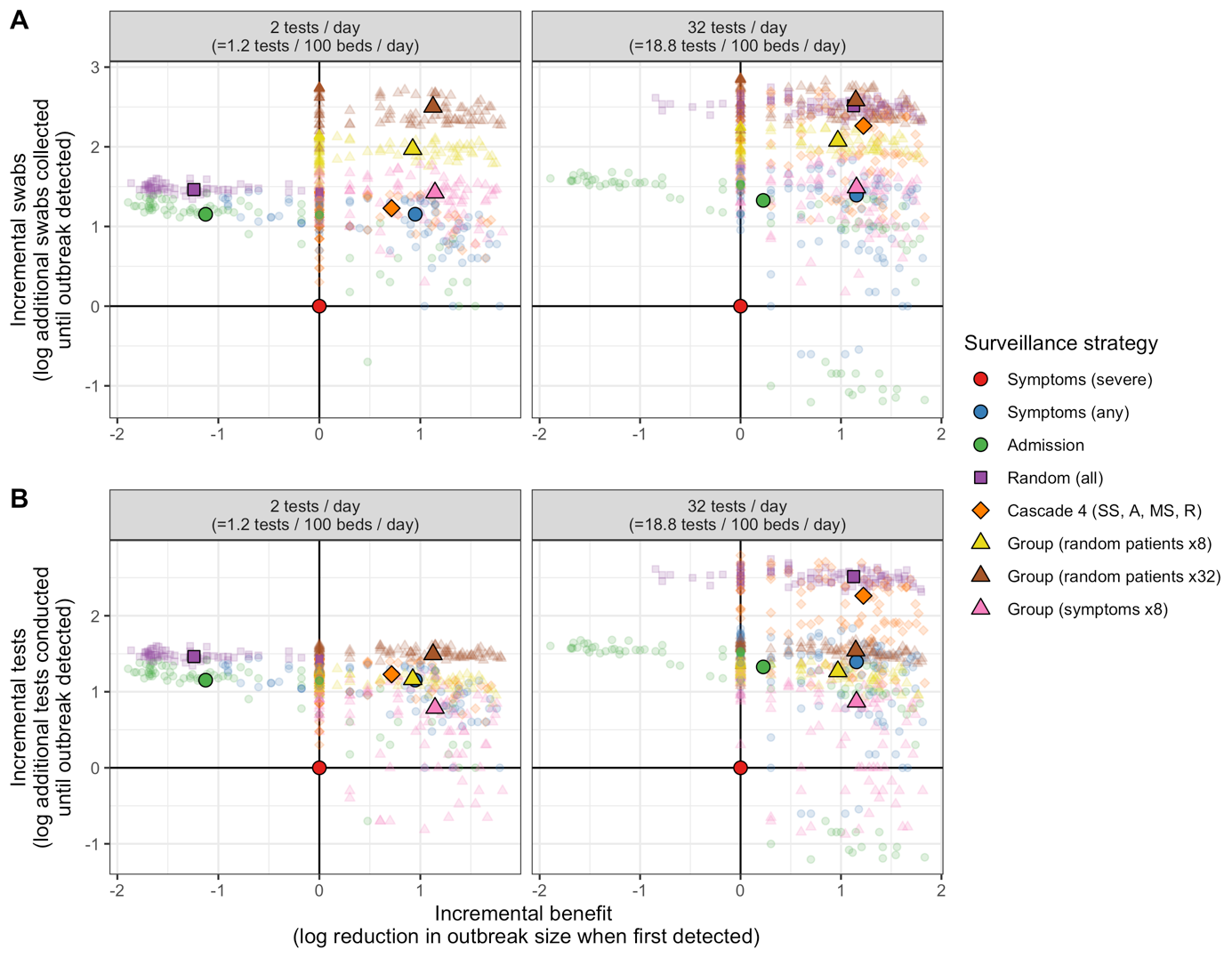


**Figure S12.** Log_10_-transformed incremental efficiency plots for simulations in the baseline LTCF with a low transmission rate (*p*=0.07%), comparing incremental resource-use in terms of nasopharyngeal swabs used (**A**) and RT-PCR tests conducted (**B**), and varying testing capacity from 2 tests/day (left panels) and 32 tests/day (right panels). Small translucent points represent median outcomes across 100 surveillance simulations for each simulated outbreak, and larger opaque points represent the mean of all medians.

**
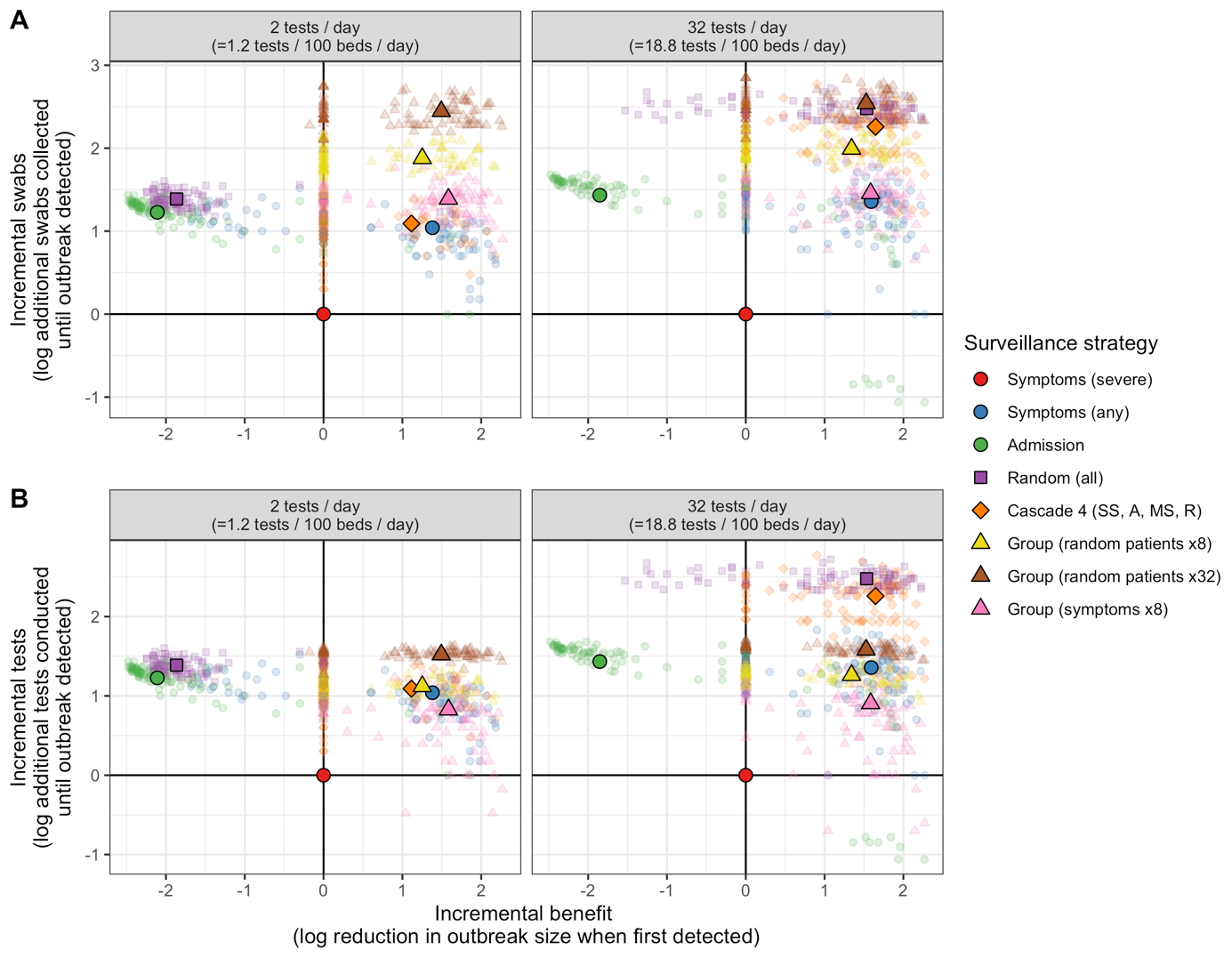
**

**Figure S13.** Log_10_-transformed incremental efficiency plots for simulations in the baseline LTCF with a high transmission rate (*p*=0.28%), comparing incremental resource-use in terms of nasopharyngeal swabs used (**A**) and RT-PCR tests conducted (**B**), and varying testing capacity from 2 tests/day (left panels) and 32 tests/day (right panels). Small translucent points represent median outcomes across 100 surveillance simulations for each simulated outbreak, and larger opaque points represent the mean of all medians.


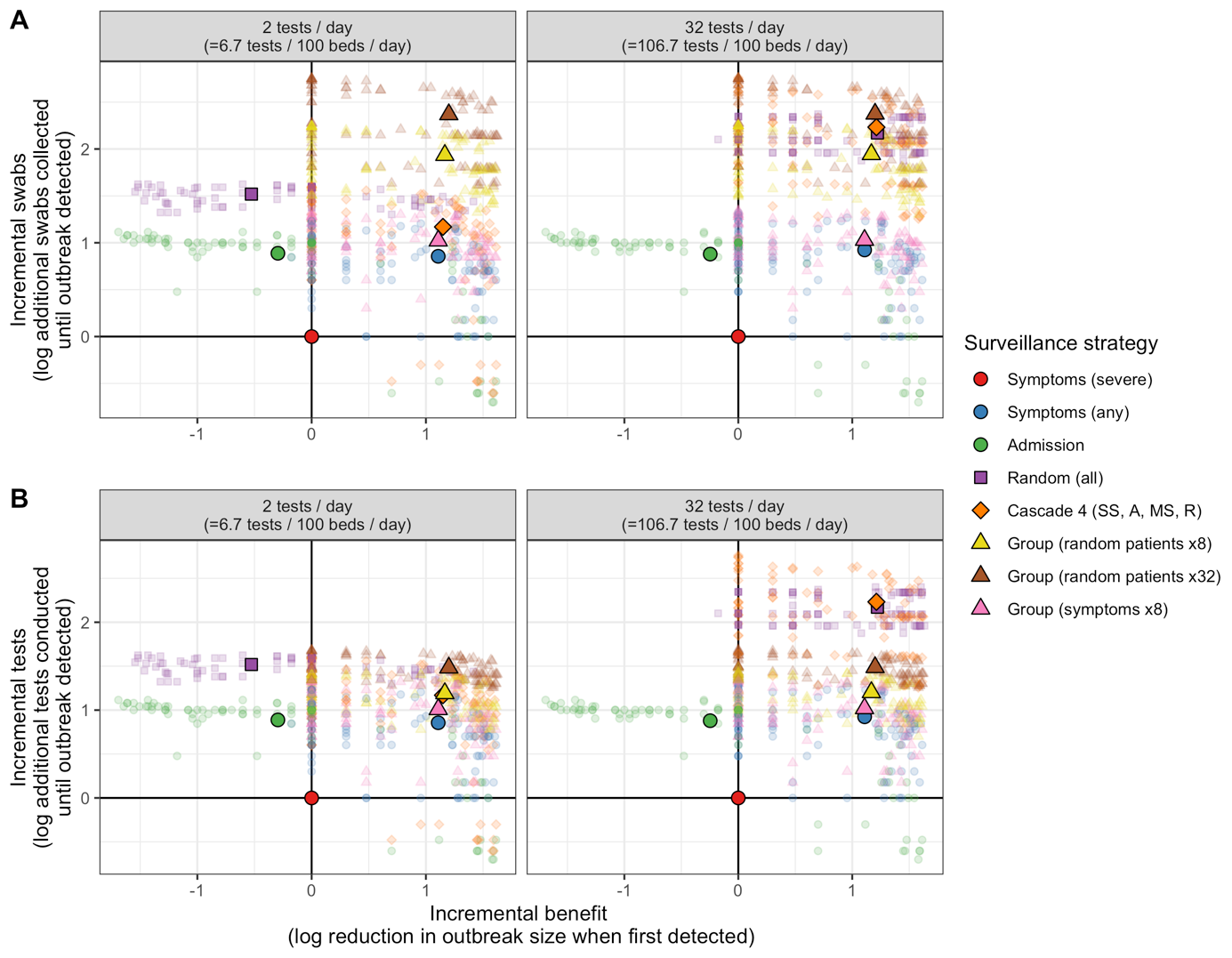


**Figure S14.** Log_10_-transformed incremental efficiency plots for simulations in the 30-bed geriatric LTCF, comparing incremental resource-use in terms of nasopharyngeal swabs used (**A**) and RT-PCR tests conducted (**B**), and varying testing capacity from 2 tests/day (left panels) and 32 tests/day (right panels). Small translucent points represent median outcomes across 100 surveillance simulations for each simulated outbreak, and larger opaque points represent the mean of all medians.


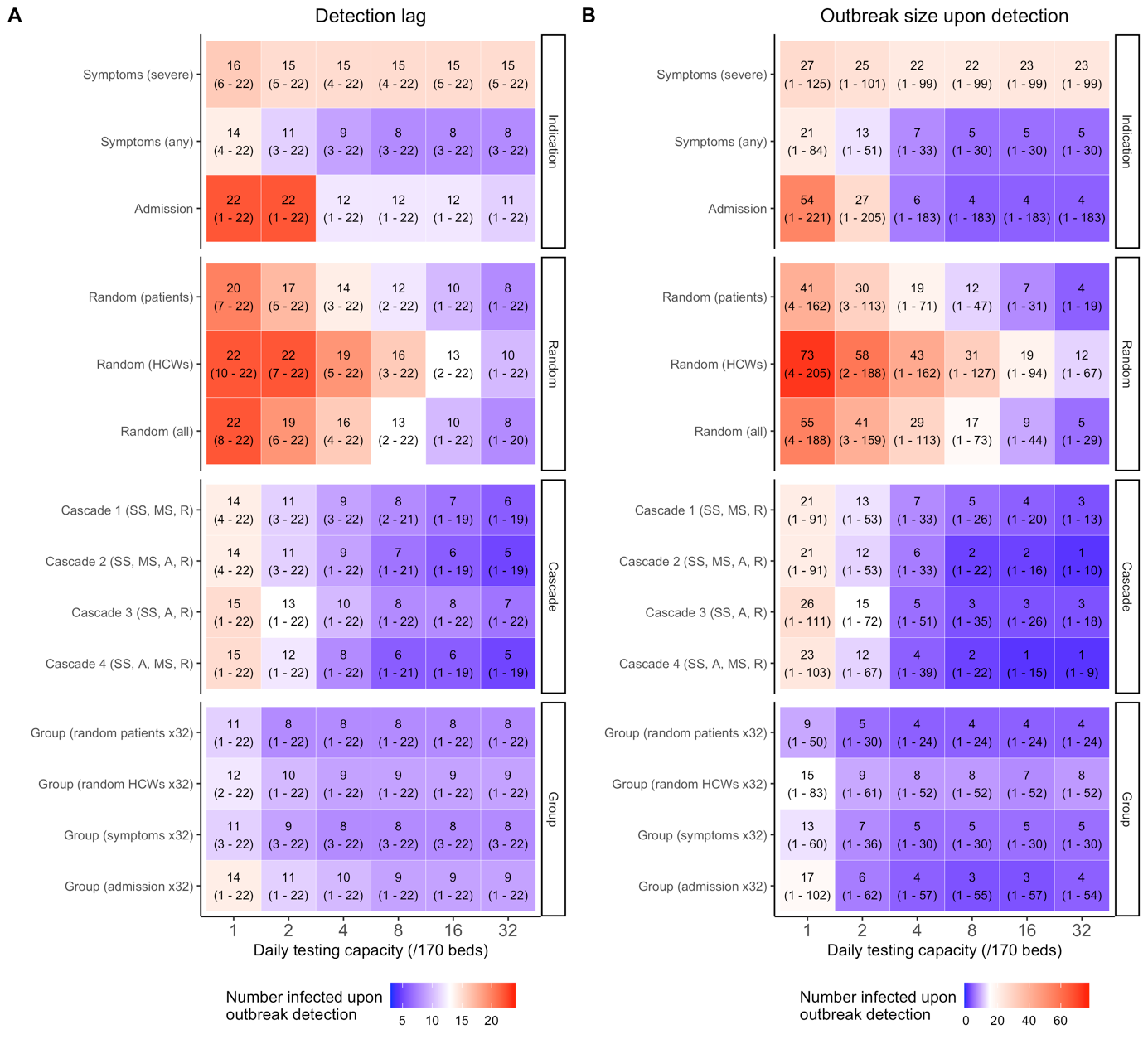


**Figure S15.** For simulations in the baseline LTCF with higher and more stable RT-PCR sensitivity (30% when exposed to SARS-CoV-2, 90% when infectious) **(A)** median lags to outbreak detection (95% uncertainty interval) and **(B)** corresponding outbreak sizes upon detection are shown for each surveillance strategy (y-axis) as a function of the daily testing capacity (x-axis). Group testing strategies assume a maximum of 32 swabs per test.
